# A brain-specific angiogenic mechanism enabled by tip cell specialization

**DOI:** 10.1038/s41586-024-07283-6

**Published:** 2024-04-03

**Authors:** Giel Schevenels, Pauline Cabochette, Michelle America, Arnaud Vandenborne, Line De Grande, Stefan Guenther, Liqun He, Marc Dieu, Basile Christou, Marjorie Vermeersch, Raoul F. V. Germano, David Perez-Morga, Patricia Renard, Maud Martin, Michael Vanlandewijck, Christer Betsholtz, Benoit Vanhollebeke

**Affiliations:** 1 Laboratory of Neurovascular Signaling, Department of Molecular Biology, ULB Neuroscience Institute, Université libre de Bruxelles (ULB), Gosselies, Belgium; 2 Max Planck Institute for Heart and Lung Research, ECCPS Bioinformatics and Deep Sequencing Platform, Bad Nauheim, Germany; 3 Department of Immunology, Genetics and Pathology, Rudbeck Laboratory, Uppsala University, Uppsala, Sweden; 4 Mass Spectrometry Facility (MaSUN), University of Namur, Namur, Belgium; 5 Center for Microscopy and Molecular Imaging (CMMI), Université libre de Bruxelles (ULB), Gosselies, Belgium; 6 Department of Medicine (Huddinge), Karolinska Institutet, Huddinge, Sweden

**Keywords:** Blood-brain barrier, Angiogenesis

## Abstract

Vertebrate organs require locally adapted blood vessels^1,2^. The gain of such organotypic vessel specializations is often deemed to be molecularly unrelated to the process of organ vascularization. Here, opposing this model, we reveal a molecular mechanism for brain-specific angiogenesis that operates under the control of Wnt7a/b ligands—well-known blood–brain barrier maturation signals^3–5^. The control mechanism relies on Wnt7a/b-dependent expression of Mmp25, which we find is enriched in brain endothelial cells. CRISPR–Cas9 mutagenesis in zebrafish reveals that this poorly characterized glycosylphosphatidylinositol-anchored matrix metalloproteinase is selectively required in endothelial tip cells to enable their initial migration across the pial basement membrane lining the brain surface. Mechanistically, Mmp25 confers brain invasive competence by cleaving meningeal fibroblast-derived collagen IV α5/6 chains within a short non-collagenous region of the central helical part of the heterotrimer. After genetic interference with the pial basement membrane composition, the Wnt–β-catenin-dependent organotypic control of brain angiogenesis is lost, resulting in properly patterned, yet blood–brain-barrier-defective cerebrovasculatures. We reveal an organ-specific angiogenesis mechanism, shed light on tip cell mechanistic angiodiversity and thereby illustrate how organs, by imposing local constraints on angiogenic tip cells, can select vessels matching their distinctive physiological requirements.

## Abstract (web summary)

A molecular mechanism for brain-specific angiogenesis operates under the control of Wnt7a/b ligands.

## Main

In vertebrates growing beyond the limit of oxygen diffusion, a vascular system branches through the body to supply nutrients and oxygen, remove cellular waste products, allow immune cell transport and support coagulation^1,6^. Contrasting with the apparent uniformity of their ancestral functions, blood vessels exhibit considerable phenotypic heterogeneity. In particular, the inner lining of blood vessels is made of highly malleable endothelial cells (ECs) that engage in a variety of two-way communications with local microenvironments^1,2,7^. By adopting organ-specific structural and molecular profiles, ECs have essential roles in organ development, metabolism, regeneration and repair. As angiogenesis is almost invariably initiated by an environmental trigger common to all expanding organs, that is, the shortage of oxygen, the cellular and molecular logic of blood vessel branching through sprouting angiogenesis and organotypic endothelial adaptations are generally viewed as distinct events, regulated by independent molecular machineries.

The central nervous system (CNS) offers a notable exception to this rule. From early developmental stages onwards, its functional complexity requires isolation and protection from peripheral chemical variations and toxins. Embryonic cerebral ECs therefore mature into a neuroprotective blood–brain barrier (BBB) phenotype, an archetypical example of EC specialization, that is initiated by endothelial Wnt–β-catenin signalling^3–5^. In response to Wnt ligands, CNS ECs reduce paracellular permeability through tight junctions, repress transcytosis, express numerous solute transporters such as GLUT1 and recruit pericytes. While, as in any other organ, brain angiogenesis is VEGF-dependent, the BBB-inductive Wnt–β-catenin signalling pathway is also required for brain angiogenesis, thereby making a direct, yet enigmatic, link between organ vascularization and organotypic endothelial specialization.

Through this coupling mechanism, no leaky blood vessels penetrate the delicate neuroepithelium, accounting for an efficient endothelial quality-control mechanism. However, how Wnt signalling impinges on the angiogenic cascade selectively in the brain and, thereby, more generally, how organs can ensure that their perfusing vessels match their specific metabolic requirements remain to be determined.

## Brain-specific angiogenesis control

**Fig. 1:**
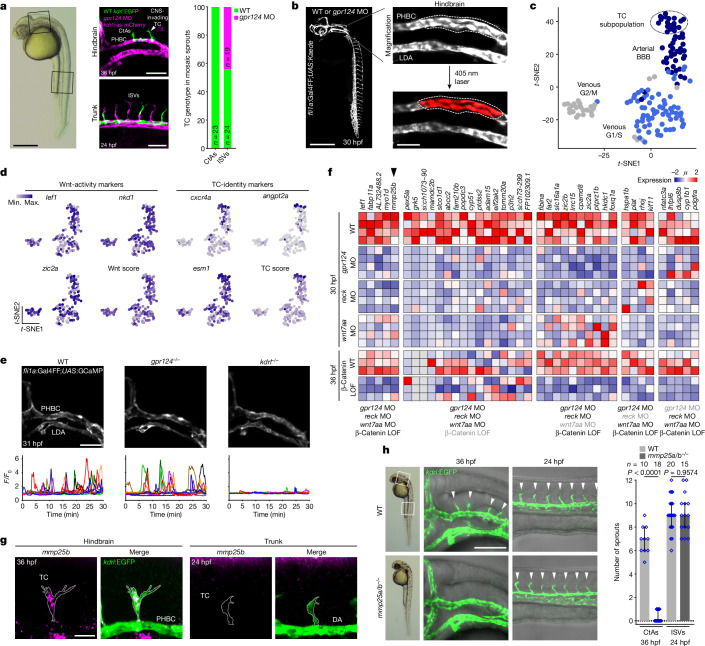
Mmp25 as a regulator of brain-specific angiogenesis. **a**, The TC genotype in mosaic sprouts during brain vascular invasion (36 hpf, *n* = 23 sprouts) and trunk ISV formation (24 hpf, *n* = 43 ISVs) of embryos obtained by five transplantation experiments of WT *kdrl:EGFP *donor cells into *gpr124* MO-injected *kdrl:ras-mCherry* hosts. **b**, In vivo photoconversion design of pre-angiogenic PHBCs. LDA, lateral dorsal aorta. **c**, *t*-Distributed stochastic neighbour embedding (*t*-SNE) analysis of PHBC EC clusters. **d**, *t-*SNE expression profiles of Wnt–β-catenin target genes and TC markers. Max., maximum; min., minimum. **e**, Time-lapse recordings of calcium oscillations in *Tg(fli1a:Gal4FF);(UAS:GCaMP7a)* PHBCs (31 to 31.5 hpf). **f**, Wnt-dependent transcripts in 30 hpf PHBCs or 36 hpf CtAs (β-catenin LOF, IWR-1 treatment). Grey labels below the heat map indicate conditions in which candidate genes are not statistically downregulated. *μ*, mean expression. **g**, Fluorescent *mmp25b* WISH and anti-EGFP staining of *Tg(kdrl:EGFP)* embryos. DA, dorsal aorta. **h**, Angiogenic sprouts (arrowheads) in the hindbrain and trunk region of *Tg(kdrl:EGFP)* embryos. *n* ≥ 10 embryos from 4 independent experiments. Data are median ± interquartile range. *P* values were calculated using nonparametric two-tailed Mann–Whitney *U*-tests. Scale bars, 400 μm (**a** (left) and **b** (left)), 100 μm (**a** (right), **e** and **h**), 50 μm (**b** (right)) and 20 μm (**g**). Source Data

**Extended Data Fig. 1:**
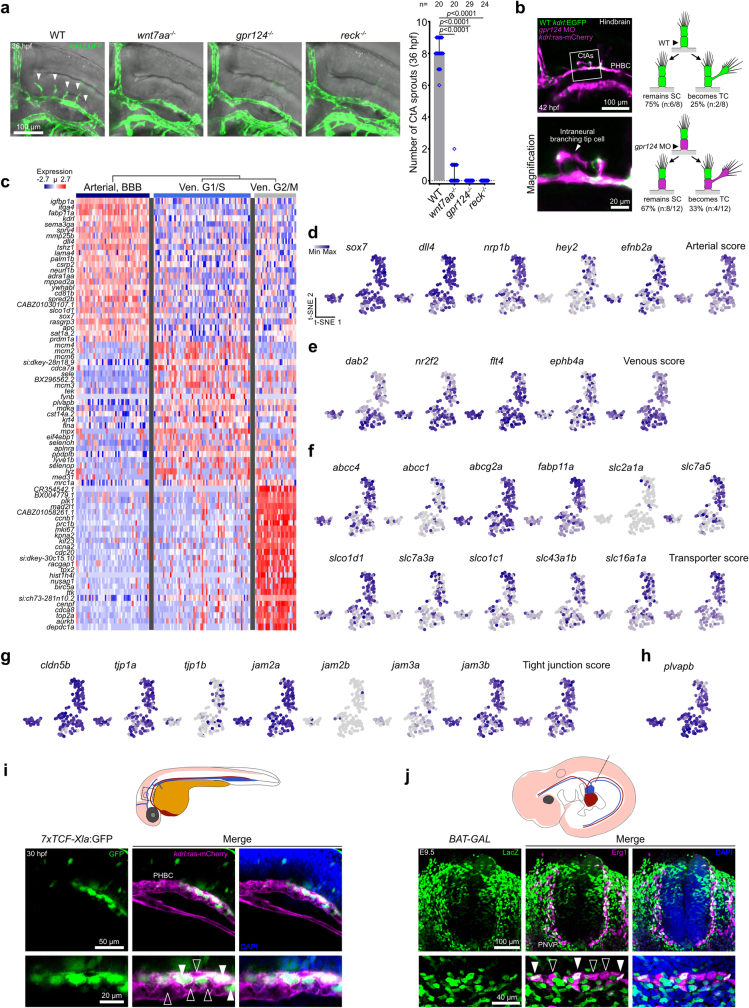
Characterization of the pre-angiogenic perineural endothelium. **a**, Lateral views and quantifications of angiogenic sprouts in the 36 hpf hindbrain of WT, *wnt7aa*^*−/−*^, *gpr124*^*−/−*^, and *reck*^*−/−*^ *Tg(kdrl:EGFP)* embryos (n ≥ 20 embryos from 3 independent experiments, arrowheads: CtA sprouts). Data represent median ± interquartile range. p-values were determined using the non-parametric Kruskal-Wallis test. **b**, TC genotype in mosaic angiogenic sprouts during post-invasive secondary branching (42 hpf) of embryos obtained by transplanting wild-type (WT) *kdrl*:EGFP^+^ donor cells into *gpr124* morpholino-injected *kdrl:*ras-mCherry hosts. The arrowhead points at a TC within the hindbrain. n = number of analysed mosaic vessels from 10 embryos recorded in 5 independent transplantation experiments. **c**, Heat map of scRNA-Seq transcriptome analysis of 30 hpf PHBC ECs showing the expression levels of the 25 highest ranked (lowest p-value) transcripts for each cell cluster. Ven: venous. **d-h**, t-SNE expression profiles of arterial markers (**d**), venous markers (**e**), BBB-associated transporters (**f**), tight junction proteins (**g**), and *plvapb*, a transcytosis marker (**h**). **i**, Lateral view of a WT *Tg(7xTCF-Xla.Siam:GFP);(kdrl:ras-mCherry)* embryo at 30 hpf, counterstained with DAPI. The solid and open arrowheads point at some *7xTCF:*GFP^+^ and *7xTCF:*GFP^−^ PHBC ECs, respectively. **j**, Immunostaining for β-Galactosidase (LacZ) and Erg1 in a transverse spinal cord section of a WT BAT-GAL E9.5 embryo, counterstained with DAPI. The solid and open arrowheads indicate some LacZ^+^ and LacZ^−^ perineural vascular plexus (PNVP) ECs, respectively. Source Data

To investigate the organotypic control of brain angiogenesis by endothelial Wnt–β-catenin signalling, we recorded this process using time-lapse confocal microscopy in genetically mosaic zebrafish embryos. As in mice^8–10^, zebrafish brain vascularization is controlled by neural-derived Wnt7a/b ligands, recognized by the atypical Gpr124–Reck complex of perineural ECs^11–14^ (Extended Data Fig. 1a). Mid-blastula transplantation of wild-type (WT) *Tg(kdrl:EGFP)* cells into *gpr124* morpholino (MO)-injected *Tg(kdrl:ras-mCherry)* hosts resulted in mosaic perineural primordial hindbrain channels (PHBCs) from which central artery (CtA) sprouts invariably invaded the brain with a WT cell at the tip cell (TC) position^11^ (Fig. 1a and Supplementary Video 1). By contrast, peripheral intersegmental vessels (ISVs) were led at equal frequencies by WT or *gpr124*-morphant TCs (Fig. 1a). Similarly, after the CNS-invasive step, *gpr124*^−^ TCs guiding secondary intraneural vessels could readily be detected (Extended Data Fig. 1b and Supplementary Video 2). Wnt–β-catenin signalling therefore appears to be selectively required in perineural TCs during the initial event of brain invasion. Within this narrow spatiotemporal window, Wnt signalling could control either TC identity or TC behaviour.

To address this, we transcriptionally profiled fluorescence-activated cell sorting (FACS)-isolated 30 hours post-fertilization (30 hpf) PHBC WT or *gpr124* MO ECs after in vivo photoconversion of *Tg(fli1a:Gal4FF);(UAS:Kaede)* transgenic embryos (Fig. 1b). Single-cell RNA-sequencing (scRNA-seq) analysis of 144 ECs from WT PHBCs revealed three major cell clusters (Fig. 1c,d and Extended Data Fig. 1c–e), namely two venous clusters (G2/M and G1/S cell cycle phases, defined by *dab2*, *nr2f2*, *flt4* and *ephb4a*) and one arterial cluster (*sox7*, *dll4*, *nrp1b*, *hey2* and *efnb2a*) containing a TC population (*cxcr4a*, *angpt2a* and *esm1*). The arterial cluster exhibited a BBB signature (Extended Data Fig. 1f–h).

**Extended Data Fig. 2:**
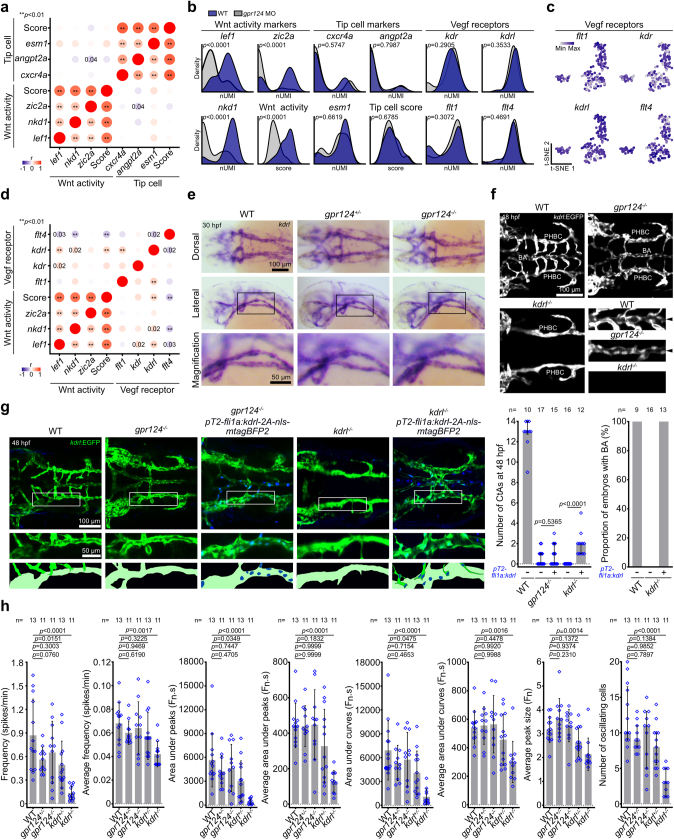
Contrasting Wnt–β-catenin and VEGF signalling functions during CNS angiogenesis. **a**, Expression correlation matrix of Wnt–β-catenin target genes and TC markers based on the scRNA-Seq transcriptome of the 30 hpf PHBC ECs. Pearson correlation coefficients (r) and p-values were calculated in R. **b**, Density plots of Wnt–β-catenin target genes, TC markers and Vegf receptors in WT and *gpr124* MO PHBC ECs. nUMI: normalized unique molecular identifier. p-values were determined using the parametric two-tailed Student’s t-test. **c**, t-SNE expression profiles of VEGF receptors within PHBC ECs. **d**, Expression correlation matrix between Wnt–β-catenin activity markers and Vegf receptor genes based on the scRNA-Seq transcriptome of the 30 hpf PHBC ECs. Pearson correlation coefficients (r) and p-values were calculated in R. **e**, Chromogenic WISH for *kdrl* in WT, *gpr124*^*+/−*^, and *gpr124*^*−/−*^ embryos at 30 hpf. **f**, Dorsal views of WT, *gpr124*^*−/−*^ and *kdrl*^*−/−*^ *Tg(kdrl:EGFP)* embryos at 48 hpf. Black arrowheads point at the basilar artery (BA). **g**, Dorsal views of 48 hpf WT, *gpr124*^*−/−*^, or *kdrl*^*−/−*^ *Tg(kdrl:EGFP)* embryos, co-injected or not at the one-cell stage with 10 pg of a *pTol2-fli1a:kdrl-2A-nls-mTagBFP2* construct and 25 pg *Tol2* transposase mRNA. The graphs show CtA numbers and proportion of embryos with a BA. n ≥ 9 embryos from 8 independent experiments. Data represent median ± interquartile range. p-values were determined using the two-tailed non-parametric Mann-Whitney test. **h**, Characterization of Ca^2+^ oscillations in WT, *gpr124*^*+/−*^, *gpr124*^*−/−*^, *kdrl*^*+/−*^, or *kdrl*^*−/−*^ *Tg(fli1a:Gal4FF);(UAS:GCaMP7a)* PHBC ECs at pre-angiogenic stages (31 to 31.5 hpf). Frequency, area under peak, area under curve, and peak size data are represented as mean ± standard deviation. p-values were determined using the parametric one-way ANOVA test. The number of Ca^2+^-oscillating cells is represented as median ± interquartile range and analysed with the non-parametric Kruskal-Wallis test. n ≥ 11 embryos from 3 independent experiments. Source Data

The pre-angiogenic PHBCs contained Wnt-positive (*lef1*, *nkd1* and *zic2a*) and Wnt-negative cells that both contributed to the venous and arterial cell clusters (Fig. 1d). This heterogeneity in Wnt signalling activity was confirmed by the analysis of zebrafish and mouse transgenic reporters (Extended Data Fig. 1i,j and Supplementary Video 3). TC and Wnt marker gene expression did not correlate across the PHBC EC population (Extended Data Fig. 2a). Wnt-independent specification of perineural TCs was confirmed by implementing a similar scRNA-seq approach in *gpr124* morphants. While, as anticipated, Wnt–β-catenin marker gene expression was strongly reduced in *gpr124* morphants, TC markers were maintained (Extended Data Fig. 2b).

Wnt–β-catenin signalling has been reported to upregulate *Vegfr2* transcripts in the postnatal retinal and brain vasculature^15^. At brain invasion stages, the expression levels of *kdrl*, the main functional homologue of mammalian *VEGFR2* in zebrafish, correlated partially with Wnt signalling activity (Extended Data Fig. 2c,d). However, *kdrl* expression did not rely on Wnt activity, as it was maintained in *gpr124* morphants (Extended Data Fig. 2b,e). Moreover, the formation of the PHBC-derived basilar artery, a *kdrl*-dependent process, was unaffected in *gpr124* mutants (Extended Data Fig. 2f), implying that Wnt-deficient PHBCs remain responsive to VEGF. Furthermore, transgenic endothelial overexpression of *kdrl* did not rescue *gpr124*^*−/−*^ phenotypes, whereas it partially corrected the control *kdrl*^*−/−*^ phenotypes (Extended Data Fig. 2g). Finally, in contrast to *kdrl*^*−/−*^ vessels, VEGF-induced cytosolic calcium oscillations^16^ were unaffected in *gpr124*^*−/−*^ *Tg(fli1a:Gal4FF);(UAS:GCaMP7a)* PHBCs (Fig. 1e, Extended Data Fig. 2h and Supplementary Video 4). Together, these data suggest that the Wnt-dependent brain-specific angiogenic defects are explained neither by defective TC specification nor by impaired VEGF signalling.

## TC angiodiversity

**Extended Data Fig. 3:**
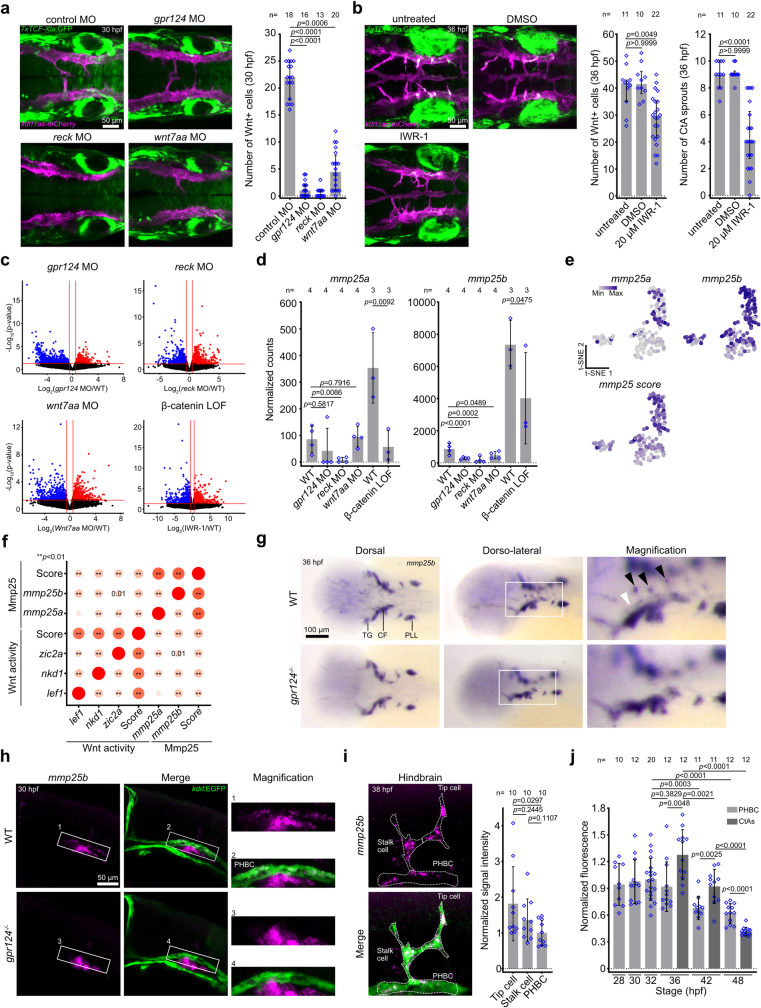
RNA-Seq analysis reveals Wnt–β-catenin-dependent expression of *mmp25a/b* in CNS ECs. **a**, Dorsal views of 30 hpf *Tg(7xTCF-Xla.Siam:GFP);(kdrl:ras-mCherry)* embryos injected with control, *gpr124, reck*, or *wnt7aa* morpholinos and quantification of *7xTCF*:GFP^+^ cells in the PHBCs. **b**, Left, dorsal views of untreated, DMSO, and IWR-1 (20 µM)-treated *Tg(7xTCF-Xla.Siam:GFP);(kdrl:ras-mCherry)* embryos at 36 hpf. Centre, number of *7xTCF*:GFP^+^ cells in the PHBCs at 36 hpf. Right, number of CtA sprouts at 36 hpf. In **a,b**, n ≥ 10 embryos from 3 independent experiments. Data represent median ± interquartile range. p-values were determined using the non-parametric Kruskal-Wallis test. **c**, Volcano plots illustrating the differential RNA-Seq expression profiles between WT and Wnt-deficient PHBC ECs. **d**, Bulk RNA-Seq normalized expression values of *mmp25a* and *mmp25b* in WT or Wnt-deficient PHBC ECs. n = brain endothelia analysed by RNA-Seq. Data represent mean ± standard deviation. In **c,d**, p-values were determined using DESeq2 analysis. **e**, scRNA-Seq t-SNE expression profile of *mmp25a* and *mmp25b* and their combined expression score in PHBC-derived EC clusters. **f**, Expression correlation matrix between Wnt–β-catenin signalling target genes and *mmp25a*, *mmp25b*, or their combined score based on the scRNA-Seq transcriptome of the 30 hpf PHBC ECs. Pearson correlation coefficients (r) and p-values were calculated in R. **g**, Dorsal and dorso-lateral views of WISH for *mmp25b* in WT and *gpr124*^*−/−*^ embryos at 36 hpf. The white arrowhead points at the PHBC, black arrowheads point at CtA TCs. TG: trigeminal nerve nuclei, CF: craniofacial nerve nuclei, PLL: posterior lateral line nuclei. **h**, Combined fluorescent in situ hybridization for *mmp25b* and anti-EGFP immunostaining of 30 hpf WT and *gpr124*^*−/−*^ *Tg(kdrl:EGFP)* embryos. The magnified view shows expression in the PHBC and the underlying CF. **i**, Combined fluorescent WISH for *mmp25b* and anti-EGFP immunostaining of *Tg(kdrl:EGFP)* embryos in 38 hpf hindbrain when CtAs harbour two cells. The normalized EC signal intensity is shown on the right. n = 10 angiogenic sprouts from 10 embryos examined over ≥ 3 independent experiments. **j**, Normalized *mmp25b* fluorescence in PHBC and CtAs during embryogenesis. n ≥ 10 embryos examined from ≥ 3 independent experiments. In **i,j**, Data represent mean ± SD. p-values were determined using the parametric two-tailed Student’s t-test. Source Data

To identify the angiogenic effectors of Wnt signalling, we performed bulk RNA-seq analysis of 30 hpf laser-photoconverted FACS-isolated PHBC ECs in WT as well as *gpr124*-, *reck-* and *wnt7aa-*morphant embryos, three Wnt–β-catenin loss of function (LOF) conditions (Extended Data Fig. 3a) that result in avascular brains^11,12,17^. We also analysed the effect of Wnt inhibition at later stages (36 hpf) by exposing embryos to IWR-1 (β-catenin LOF) from 26 hpf onwards (Extended Data Fig. 3b). This strategy identified 40 genes of which the expression was significantly downregulated in at least three Wnt LOF conditions (Fig. 1f and Extended Data Fig. 3c). Among these candidate genes, known Wnt-target genes were found (*lef1*, *fabp11a*, *slc16a1a*, *zic2a* and *zic2b*), validating the approach. Five genes were downregulated in all four conditions, including *mmp25b*, one of the two zebrafish *mmp25* paralogues (Fig. 1f and Extended Data Fig. 3d). Low-expressed *mmp25a* was also downregulated in two Wnt-LOF conditions (Extended Data Fig. 3d). Both paralogues were enriched in the arterial TC cluster (Extended Data Fig. 3e) and correlated with Wnt activity markers (Extended Data Fig. 3f).

Besides in trigeminal, craniofacial and posterior lateral line ganglia^18^, *mmp25b* transcripts were detected by chromogenic whole-mount in situ hybridization (WISH) in scattered cells of the 36 hpf hindbrain anatomically compatible with PHBCs and CtA TCs (Extended Data Fig. 3g). The signal in the presumptive ECs was *gpr124* dependent, whereas the signal in sensory ganglia was not (Extended Data Fig. 3g). Combined fluorescence WISH and immunofluorescence staining confirmed the EC- and *gpr124*-dependent nature of the signal (Extended Data Fig. 3h). Notably, *mmp25b* transcripts were detected in hindbrain TCs but were absent in ISV TCs (Fig. 1g). In the hindbrain, *mmp25b* expression was higher in TCs compared with in stalk cells (SCs) and PHBC phalanx cells (Extended Data Fig. 3i). Consistent with a potential role in brain vascular invasion, *mmp25b* expression peaked in 36 hpf CtAs, decreasing thereafter in PHBC and CtAs (Extended Data Fig. 3j).

**Extended Data Fig. 4:**
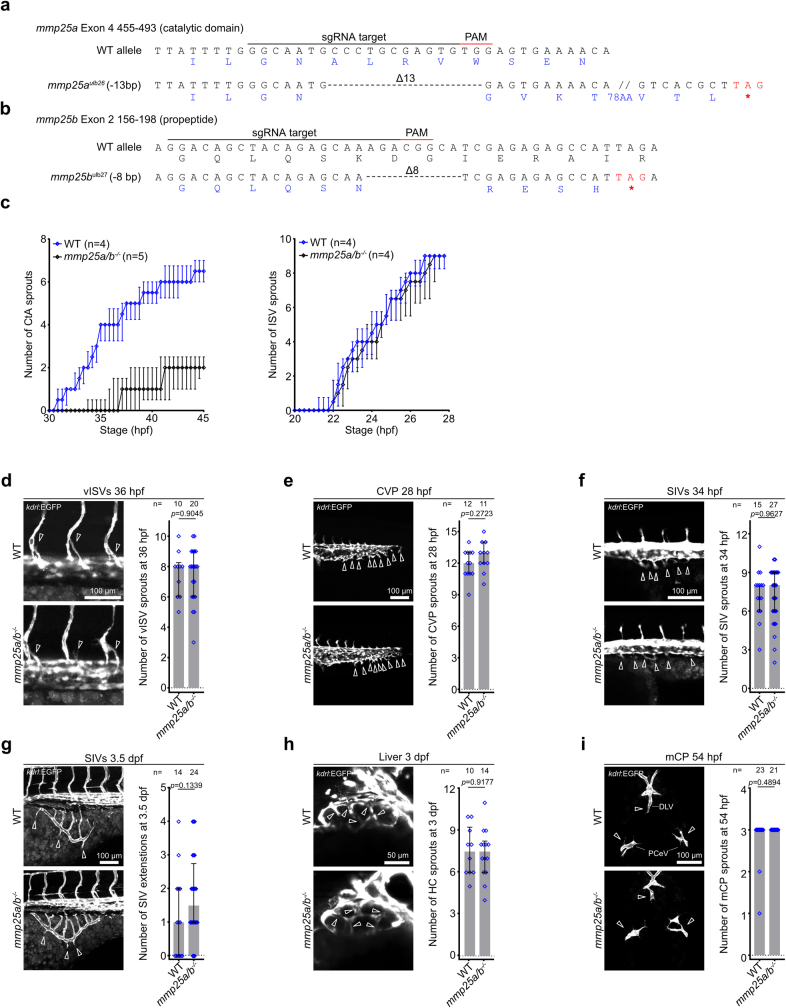
Mmp25 mutagenesis and organotypic requirement in zebrafish blood vessels. **a**, Sequences of WT *mmp25a* and the *mmp25a*^*ulb26*^ mutant allele. **b**, Sequences of WT *mmp25b* and the *mmp25b*^*ulb27*^ mutant allele. **c**, Time-course analysis of the number of CtA and ISV angiogenic sprouts on one side of WT and *mmp25a/b*^*−/−*^ embryos (n ≥ 4 embryos from 3 independent experiments). **d-i**, Comparison of the developing vasculature in WT and *mmp25a/b* mutant *Tg(kdrl:EGFP)* zebrafish (n ≥ 10 embryos or larvae from 3 independent experiments) during venous intersegmental vessel sprouting (vISV) at 36 hpf (**d**), caudal vein plexus angiogenesis (CVP) at 28 hpf (**e**), sub-intestinal vein (SIV) sprouting at 34 hpf (**f**) and 3.5 dpf (**g**), liver vascularization at 3 dpf (**h**), and formation of the myelencephalic choroid plexus (mCP) irrigating vessels at 54 hpf (**i**). DLV: dorsal longitudinal vein. PCeV: posterior cerebral vein. Arrowheads point at angiogenic sprouts. In all panels, data represent median ± .two-tailed Mann-Whitney test. Source Data

**Extended Data Fig. 5:**
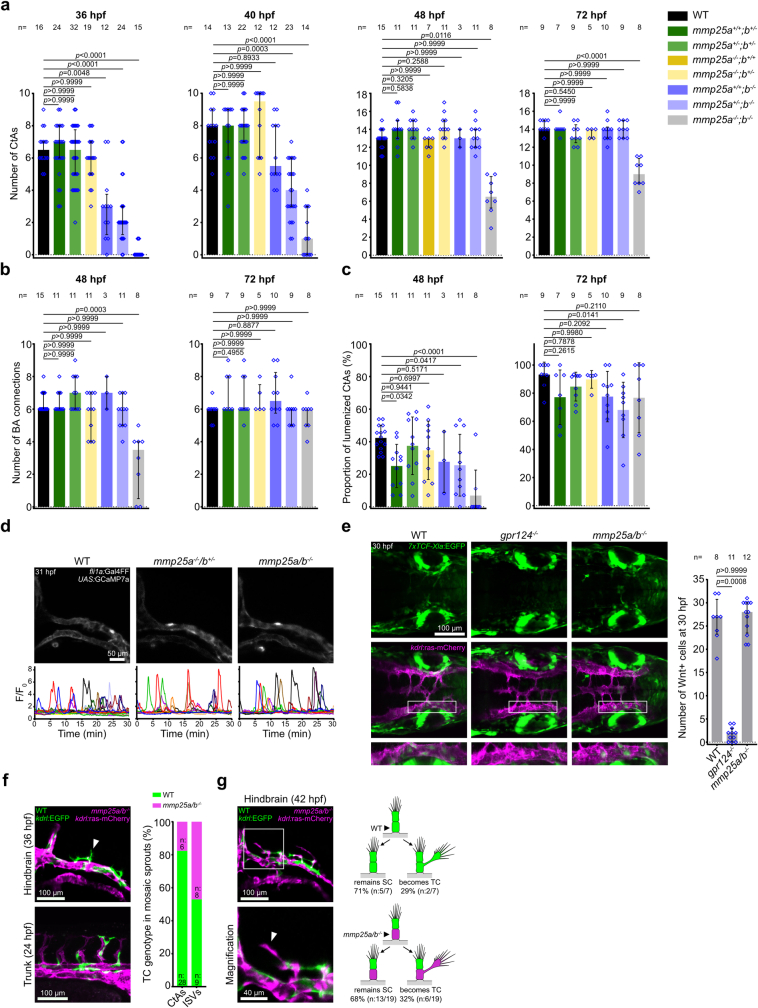
Phenotypic characterization of zebrafish *mmp25a/mmp25b* mutants. **a**, Number of CtAs in 36, 40, 48, and 72 hpf embryos and larvae with different combinations of mutant *mmp25a* and *mmp25b* alleles. **b**, Number of connections to the basilar artery (BA) at 48 and 72 hpf. Data represent median ± interquartile range. p-values were determined using the non-parametric Kruskal-Wallis test. **c**, Percentage of lumenized CtAs at 48 and 72 hpf. Data represent mean ± standard deviation. p-values were determined using the parametric one-sided ANOVA. For **a-c**, n = embryos analysed from ≥ 3 independent experiments. **d**, Lateral confocal images and quantification of normalized fluorescence (F/F_0_) of WT, *mmp25a*^*−/−*^*/b*^*+/−*^, and *mmp25a/b*^*−/−*^ *Tg(fli1a:Gal4FF);(UAS:GCaMP7a)* PHBC ECs at pre-angiogenic stages (31 to 31.5 hpf). For each genotype, images and GCaMP intensity profiles are representative of ≥ 6 embryos recorded from 4 independent experiments. **e**, Dorsal views of 30 hpf WT, *gpr124*^*−/−*^, and *mmp25a/b*^*−/−*^ *Tg(7xTCF-Xla.Siam:GFP);(kdrl:ras-mCherry)* embryos (n ≥ 8 embryos from 3 independent experiments). The graph shows the number of *7xTCF*:GFP^+^ PHBC ECs at 30 hpf. Data represent median ± interquartile range. p-values were determined using the non-parametric Kruskal-Wallis test. **f**,**g**, Lateral views of angiogenic sprouts in the hindbrain at the initial step of brain vascular invasion (**f**, 36 hpf) or at later intraneural branching steps (**g**, 42 hpf) and in the trunk region (**f**, 24 hpf) of genetically mosaic embryos obtained by blastula-stage transplantation of wild-type (WT) *kdrl*:EGFP^+^ donor cells into *mmp25a/b*^*−/−*^ *kdrl:*ras-mCherry hosts. White arrowheads point at TCs within the hindbrain. For **f** and **g**, n = distinct mosaic vessels from 8 embryos recorded in 3 independent transplantation experiments. Source Data

**Extended Data Fig. 6:**
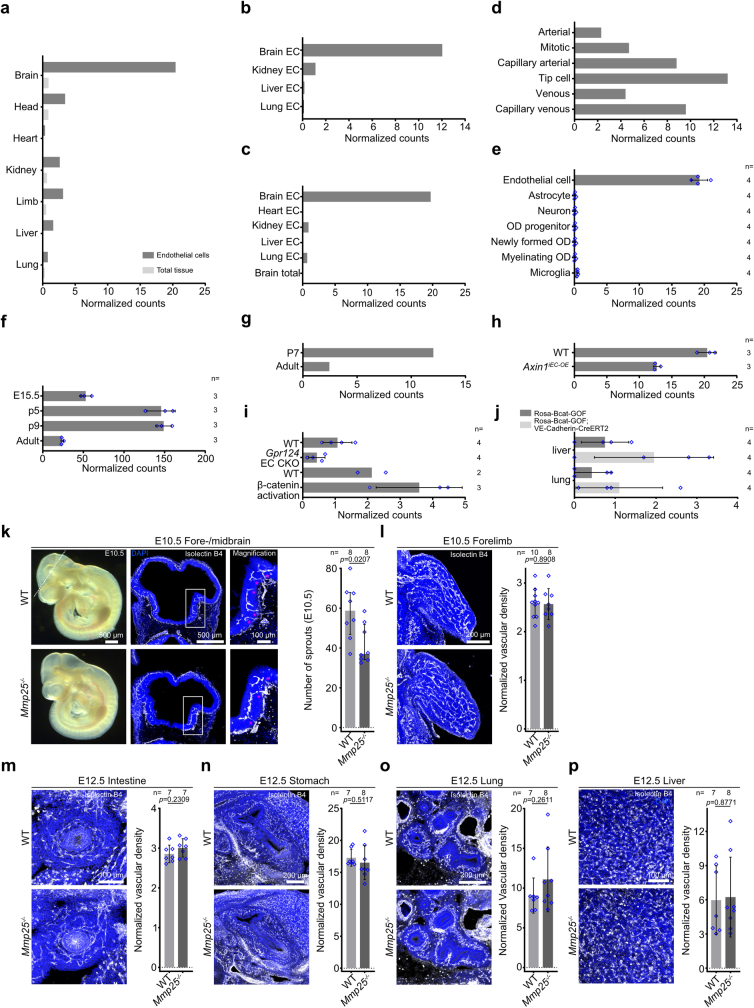
*Mmp25* expression and organotypic angiogenic requirement in mice. **a-j**, Normalized mouse *Mmp25* expression levels in *Cdh5*^*+*^ ECs and whole organ extracts at E14.5 (**a**)^77^, ECs from different organs (*Tie2*^*+*^) at p7 (**b**)^78^, *Cdh5*^*+*^ adult ECs from various organs (**c**)^79^, single brain *Tie2*^*+*^ ECs at p7 (**d**)^78^, different cell types in the p7 cortex (**e**)^80^, brain ECs (*CD31*^+^; *CD45*^*−*^) at E15.5, p5, p9, and in adults (**f**)^44^, p7 and adult *Tie2*^*+*^ brain ECs (**g**)^78^, brain ECs (*CD31*^*+*^*)* of control (*Cdh5CreERT2;mCherryTRAP)* or *Axin1*^*iEC-OE*^ (*Axin1*^*+/+*^*; Cdh5CreERT2;mCherryTRAP)* mice at E14.5 (**h**)^81^, adult brain ECs (*CD31*^*+*^) of control (*Gpr124*^flox/+^;*ROSA-CreER)*, *Gpr124* CKO (*Gpr124*^flox/−^;*ROSA-CreER)*, control (*Ctnnb1*^*+/+*^*; Cdh5-CreER*), and β-catenin activated (*Ctnnb1*^*lox(ex3)/+*^; *Cdh5-CreER*) mice (**i**)^82^, and adult liver and lung ECs (*Cdh5*^*+*^*)* of control (*Rosa-Bcat-GOF*) and with β-catenin activated (*Rosa-Bcat-GOF; Cdh5-CreERT2*) mice (**j**)^79^. Data represent mean ± standard deviation. In **e,f,h-j**, n = number of animals analysed by RNA-seq. **k**, Isolectin B4 staining of blood vessels on sections (dashed lines) through the E10.5 WT and *Mmp25*^*−/−*^ mouse fore- and midbrain. DAPI: 4′,6-diamidino-2-phenylindole. Magenta asterisks indicate CNS-penetrating angiogenic sprouts. Quantifications are provided on the right. Data represent median ± interquartile range, and p-values were determined using the non-parametric two-tailed Mann-Whitney test. **l-p**, Comparison of the vascular networks of WT and *Mmp25* KO mice in the E10.5 forelimb buds (**l**), and the E12.5 intestinal wall (**m**), gastric wall (**n**), lung buds (**o**) and liver (**p**). Data represent mean ± SD. p-values were determined using the parametric two-tailed Student’s t-test. In **k-p**, n ≥ 7 embryos from 3 independent experiments. Source Data

Also known as leukolysin^19–23^, the glycosylphosphatidylinositol (GPI)-anchored Mmp25 has not been linked to angiogenesis or Wnt signalling to date. To assess Mmp25 function, we generated *mmp25a* and *mmp25b* frameshift alleles in zebrafish using CRISPR–Cas9 (Extended Data Fig. 4a,b). Brain angiogenesis was strongly, although not completely, impaired in double homozygous *mmp25a*^*−/−*^*mmp25b*^*−/−*^ (*mmp25a/b*^*−/−*^) mutants (Fig. 1h, Extended Data Fig. 4c and Supplementary Video 5). By contrast, peripheral angiogenesis remained unaffected (Fig. 1h, Extended Data Fig. 4c–i and Supplementary Video 6). No other morphological phenotypes were detected in *mmp25a/b* mutants. Gene-dosage experiments revealed that *mmp25* paralogues exhibit partially redundant functions, with *mmp25b*^*−/−*^ phenotypes aggravated by the gradual loss of WT *mmp25a* alleles (Extended Data Fig. 5a–c). By contrast, *mmp25a*^*−/−*^ and *mmp25a*^*−/−*^*b*^*+/−*^ embryos exhibited no vascular defects, the latter of which were used as controls in subsequent experiments. Genetic inactivation of *mmp25a/b* did not affect VEGF-induced calcium oscillations or Wnt–β-catenin signalling in PHBCs (Extended Data Fig. 5d,e and Supplementary Video 7). In genetically mosaic embryos, *mmp25a/b-*mutant cells were outcompeted by WT cells for the TC position selectively in brain-invading sprouts, but not in trunk ISVs or during secondary sprouting events within the brain (Extended Data Fig. 5f,g and Supplementary Video 8). This TC autonomous requirement is reminiscent of the overall function of Wnt signalling in CNS angiogenesis, and compatible with the membrane retention of Mmp25 through a GPI anchor. Mining published datasets revealed an evolutionarily conserved expression pattern in mice, with *Mmp25* qualifying as an endothelial-enriched, brain-specific and Wnt-dependent transcript (Extended Data Fig. 6a–j). MMP25 also contributed to brain vascularization in mice, as constitutive knockouts^23^ exhibited a partial reduction in angiogenic sprouts in the embryonic day 10.5 (E10.5) midbrain and forebrain (Extended Data Fig. 6k). As in zebrafish, the mouse *Mmp25* vascular phenotypes were CNS-specific, with no defects detected in peripheral organs (Extended Data Fig. 6l–p).

## The distinctive MMP repertoire of brain TCs

**Fig. 2:**
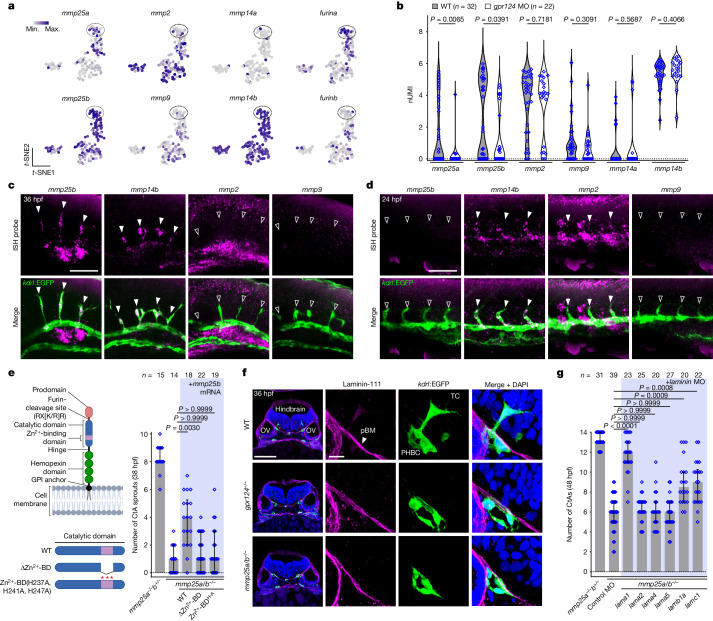
TCs require a specialized MMP repertoire to migrate across the pial basement membrane. **a**, *t*-SNE expression profiles of *mmp* and *furin* genes. **b**, Expression levels of *mmp* genes in WT and *gpr124-*MO PHBC ECs. Dots represent individual cells. nUMI, normalized unique molecular identifier. *P* values were calculated using parametric two-tailed Student’s *t*-tests. **c**,**d**, Fluorescent *mmp25b*, *mmp14b*, *mmp2* or *mmp9* WISH and anti-EGFP staining of *Tg(kdrl:EGFP)* embryos in the hindbrain (**c**) and trunk region (**d**). The solid and open arrowheads label *mmp*-positive and -negative sprouts, respectively. **e**, CtA sprouts in 38 hpf *Tg(kdrl:EGFP)* embryos (*n* ≥ 14 embryos from ≥3 independent experiments), injected at the one-cell stage with 200 pg of *mmp25b* mRNA or its variants (left). BD, binding domain; H-A, H237A, H241A, H247A. The diagram was created using BioRender. **f**, Anti-laminin-111 immunofluorescence staining of transverse hindbrain sections, counterstained with DAPI. OV, otic vesicle. **g**, Hindbrain CtAs in *Tg(kdrl:EGFP)* embryos (*n* ≥ 20 embryos from ≥3 independent experiments) injected with control or laminin MOs. In **e** and **g**, data are median ± interquartile range. *P* values were calculated using nonparametric Kruskal–Wallis tests. Scale bars, 100 μm (**f** (left)), 50 μm (**c** and **d**) and 10 μm (**f** (right)). Source Data

Vertebrate genomes encode numerous MMPs, some of which are expressed by ECs. Mmp14 (also known as MT1-MMP) in particular is a well-known TC-enriched angiogenic effector^24–27^ that, after TIMP2-dependent activation of Mmp2, amplifies the proteolytic activity of migrating cells^24,25,28–30^. While peripheral TCs exhibited the anticipated MMP repertoire (*mmp14b*^*+*^*mmp2*^+^), brain TCs lacked *mmp2* transcripts, expressing *mmp25b* instead (Fig. 2a–d). In comparison to *mmp25b*, the expression of *mmp14b* appeared uniform across PHBC ECs (Fig. 2a) and was Wnt–β-catenin independent (Fig. 2b). *mmp9* was expressed at low levels in ECs.

**Extended Data Fig. 7:**
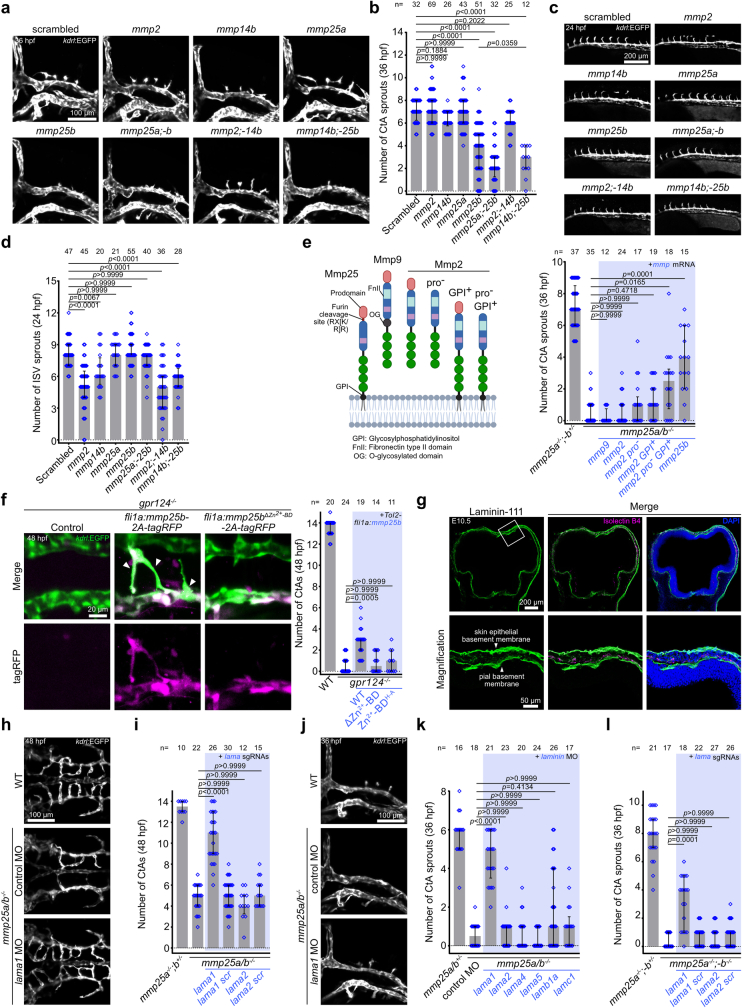
Genetic interaction between *mmp25* and pial basement membrane components during brain angiogenesis. **a-d**, Lateral views and quantification of 36 hpf hindbrain CtAs (**a,b**) or 24 hpf trunk ISVs (**c,d**) in WT *Tg(kdrl:EGFP)* embryos injected at the one-cell stage with 150 pg of *zCas9* mRNA and 60 pg of the illustrated sgRNAs. **e**, Left, schematics of Mmp25, Mmp9 and Mmp2, and its variants. Right, quantification of CtA sprouts in 36 hpf *mmp25a/b* mutant embryos, injected at the one-cell stage with 200 pg of the indicated mRNA. The diagram was created using BioRender. **f**, Transgenic *fli1a*-driven endothelial expression of an active or inactive form of Mmp25 in *gpr124*^*−/−*^ *Tg(kdrl:EGFP)* embryos. Mmp25 and its variant are expressed as P2A-tagRFP fusions. Quantifications of 48 hpf CtAs (arrowheads) are shown on the right. **g**, Immunostaining of laminin-111 on a section through the E10.5 mouse forebrain and midbrain, counterstained with Isolectin B4 and DAPI. **h**, Dorsal views of 48 hpf *Tg(kdrl:EGFP) mmp25* mutant embryos injected at the one-cell stage with a control or a *lama1*-targeting morpholino. **i**, Quantification of hindbrain CtAs in 48 hpf embryos, injected at the one-cell stage with 150 pg of *zCas9* mRNA and 60 pg of the illustrated sgRNAs. **j**, Lateral views of 36 hpf *Tg(kdrl:EGFP)* embryos injected at the one-cell stage with a control or a *lama1*-targeting morpholino. **k,l**, Quantification of hindbrain CtA sprouts in 36 hpf *mmp25a/b* mutant embryos, injected at the one-cell stage with a control or laminin morpholinos (**k**), or with 150 pg of *zCas9* mRNA and 60 pg of the illustrated sgRNAs (**l**). In all panels, data represent median ± interquartile range. p-values were determined using the non-parametric Kruskal-Wallis test for multiple comparisons, and the Mann-Whitney test for the single comparison of *mmp25b* vs. *mmp25b/mmp14* in **b**. In **b,d-f,i,k,l** n ≥ 10 embryos from 3 (**b,d,i, k, l**), 5 (**e**), or 6 (**f**) independent experiments. Source Data

Reflecting these distinct expression profiles, brain angiogenesis was largely unaffected by *mmp2* or *mmp14b* genetic inactivation, while robustly reduced in *mmp25b* or *mmp25a/b* crispants (Extended Data Fig. 7a,b). However, targeting *mmp14b* led to a slight but non-significant reduction in CtAs, which was unaffected by the additional mutagenesis of *mmp2*. The combined deletion of *mmp25b* and *mmp14b* modestly aggravated the *mmp25b* phenotypes, suggesting that *mmp14b* marginally contributes to brain angiogenesis in an *mmp2*-independent manner. In peripheral ISVs, *mmp2* and *mmp14b* contributed to angiogenic sprouting, while *mmp25a* and *mmp25b* were dispensable (Extended Data Fig. 7c,d). The combined deletion of *mmp2* and *mmp14b* did not exacerbate the individual phenotypes, which is compatible with their function in an Mmp2–Mmp14 complex. In summary, the transcriptional and functional MMP repertoire of TCs differed between CtAs and ISVs.

To determine whether shifting the brain TC repertoire (*mmp25*^*+*^*mmp14*^*+*^) to a peripheral one (*mmp2*^*+*^*mmp14*^*+*^) is compatible with brain angiogenesis, we injected mRNA encoding *mmp25b*, *mmp9*, *mmp2* or variants thereof into *mmp25a/b*^*−/−*^ one-cell stage embryos. While the restoration of *mmp25b* expression partially rescued *mmp25a/b*^*−/−*^ phenotypes, *mmp2 or mmp9* did not (Extended Data Fig. 7e). Mmp2, Mmp9, and Mmp25, produced as zymogens, differ in their activation mode. While Mmp2 and Mmp9 activation occurs extracellularly by various proteases, including Mmp14^28,29^, Mmp25 activation occurs within the secretory pathway, after processing by furin-like proprotein convertases^19–21^. Notably, *furina* is highly expressed in the PHBC TC cluster, possibly accounting for a robust activation of Mmp25 (Fig. 2a). Moreover, in contrast to the secreted Mmp2 and Mmp9, Mmp25 is retained at the cell surface through a GPI anchor, which may help to concentrate the proteolytic activity^31,32^. Assessing the brain angiogenic potential of *mmp2* mRNA variants encoding a constitutively active form of the enzyme (without the prodomain (Pro^−^)), a GPI-anchored version (GPI^+^) or both together revealed that only a variant exhibiting the combination of these properties (Pro^−^GPI^+^) was competent for brain angiogenesis (Extended Data Fig. 7e).

## The pial basement membrane obstacle

Brain angiogenesis required an EC-autonomous catalytically active form of Mmp25, as revealed by mRNA (Fig. 2e) and transgenic endothelial (*fli1a* promoter) overexpression rescue experiments (Extended Data Fig. 7f). As Wnt activity, and therefore Mmp25, are selectively required during the initial step of brain vascular invasion, we reasoned that its substrate might reside within the pial (or glia limitans) basement membrane (pBM) enwrapping the developing neuroepithelium. The pBM indeed constitutes a physical barrier that prevents radial overmigration of neurons and glial cells into the meninges^33–37^. Reciprocally, it could therefore represent an obstacle for vascular ingression into the CNS.

Laminin-111 is a well-established structural component of the pBM^34,37–40^. Other laminins are found in distinct BMs, including laminin-411 and laminin-511 around blood vessels^39^. In 30 hpf zebrafish embryos and E10.5 mice, anti-laminin-111 antibodies decorated the external surface of the developing brain (Fig. 2f and Extended Data Fig. 7g), thereby defining a landmark interface through which perineural endothelial TCs must navigate, a function that is seemingly defective in *gpr124-* and *mmp25a/b*-mutant zebrafish (Fig. 2f).

**Extended Data Fig. 8:**
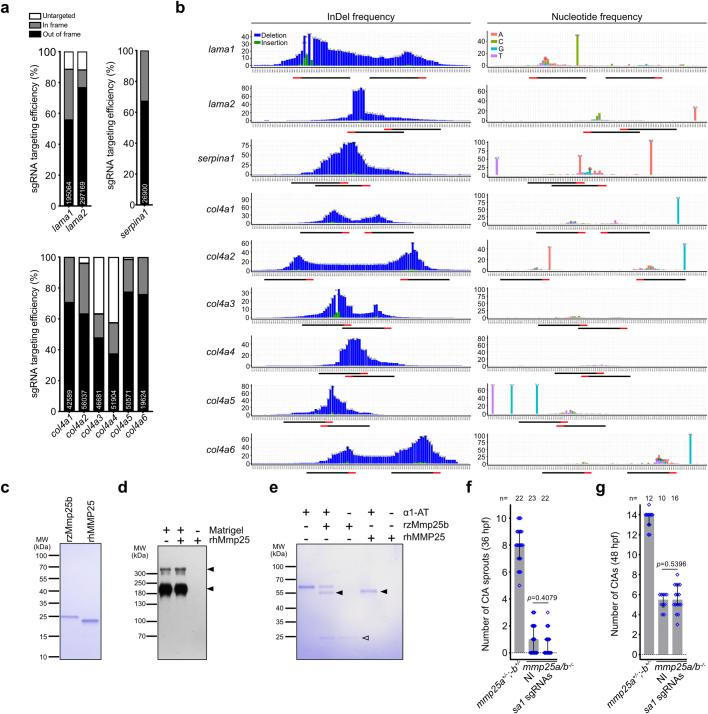
Somatic gene disruption efficiencies in zebrafish and recombinant Mmp25 catalytic domains production in *E.coli.* **a**,**b**, Targeting efficiencies (**a**) and SNPs and INDELs distributions (**b**) of embryos co-injected with 150 pg of *zCas9* mRNA and 60 pg of the indicated targeting sgRNAs, as detected by Illumina amplicon deep sequencing. **c**, Coomassie blue staining of 400 ng of recombinant zebrafish and human his-tagged Mmp25 catalytic domains produced in *E.coli*, and purified on Ni-NTA resins. rzMmp25b: recombinant catalytic domain of zebrafish Mmp25, rhMMP25: recombinant catalytic domain of human MMP25. **d**, Western blot showing laminin-111 chains (black arrowheads) in 15 µg of Matrigel, after exposure or not to rhMMP25. **e**, Coomassie blue staining of 750 ng α1-antitrypsin (α1-AT, 2 µM), after exposure to 300 ng of rzMmp25b (2 µM) or 12 ng of rhMMP25 (75 nM). The black arrowheads point to α1-AT degradation products. The open arrowhead points to rzMmp25b. rhMMP25 is below detection limit. **f**,**g**, Quantification of hindbrain CtAs of control (*mmp25a*^*−/−*^*;b*^*+/−*^) and *mmp25a/b*^*−/−*^ *Tg(kdrl:EGFP)* embryos at 36 hpf (**f**) and 48 hpf (**g**), injected or not (NI) at the one-cell stage with 150 pg of *zCas9* mRNA and 60 pg of a *serpina1* (*sa1*)-targeting sgRNA. Data represent median ± interquartile range. p-values were determined using the non-parametric two-tailed Mann-Whitney test. For **f** and **g**, n ≥ 10 embryos from 2 independent experiments. Source Data

MO- and CRISPR–Cas9-based genetic invalidation of *lama1*, *lamb1a* or *lamc1*, the three constitutive chains of laminin-111, partially rescued brain angiogenesis in *mmp25a/b*^*−/−*^ embryos, whereas interfering with *lama2*, *lama4* or *lama5* had no effect (Fig. 2g and Extended Data Figs. 7h–l and 8a,b). These observations suggest that weakening the pBM alleviates the need for Mmp25 in brain angiogenesis. The effect of laminin-111 inactivation is probably indirect, through a general destabilization of the pBM, as recombinant catalytic domains of zebrafish and human MMP25 (rzMmp25b and rhMMP25, respectively, Extended Data Fig. 8c) did not cleave laminin-111 in vitro (Extended Data Fig. 8d), as previously reported^22^. Notably, the activity of the recombinant enzymes was validated on α-1 antitrypsin (Extended Data Fig. 8e), a known substrate of which the genetic inactivation did not affect *mmp25* phenotypes (Extended Data Fig. 8a,b,f,g).

## Mmp25-substrate identification

**Fig. 3:**
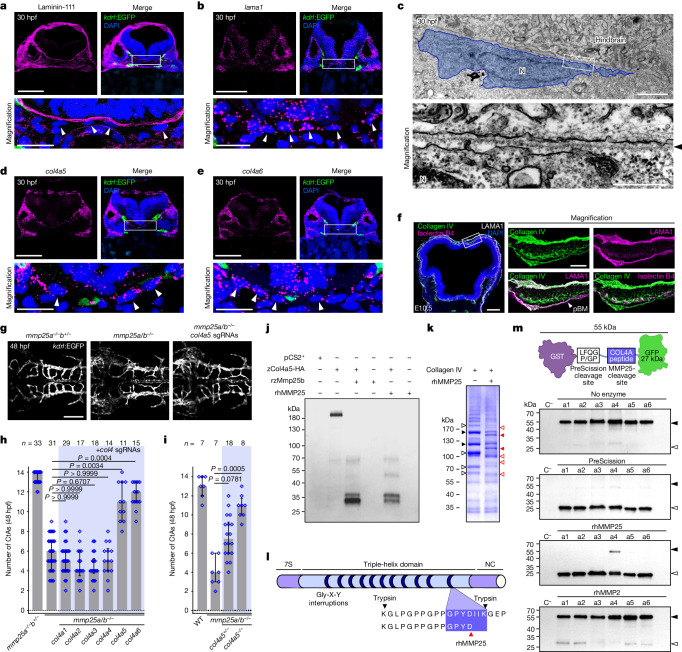
Mmp25 cleaves meningeal fibroblast-derived Col4a5/6. **a**,**b**, Laminin-111 immunostaining (**a**) and *lama1* FISH analysis and EGFP immunostaining (**b**) of *Tg(kdrl:EGFP)* transverse zebrafish hindbrain sections, counterstained with DAPI. The arrowheads indicate meningeal fibroblast nuclei. **c**, Transmission electron micrograph showing a meningeal fibroblast next to the pBM (arrowhead). N, nucleus. **d**,**e**, FISH analysis as in **b** of *col4a5* (**d**) and *col4a6* (**e**) with EGFP immunostaining. **f**, Collagen IV and laminin-111 (anti-LAMA1) co-immunostaining on a E10.5 mouse forebrain and midbrain section, counterstained with isolectin B4 and DAPI. **g**,**h**, Dorsal views (**g**) and quantification (**h**) of *Tg(kdrl:EGFP)* hindbrain CtAs in embryos (*n* ≥ 11 embryos from 3 independent experiments), injected with the illustrated sgRNAs and *zCas9* mRNA. **i**, Hindbrain CtAs in embryos (*n* ≥ 7 embryos from 4 independent experiments) crossed to the dragnet *col4a5* allele. **j**, Anti-HA western blot analysis of zCol4a5–HA-containing HEK293T cell extracts (or control pCS2^+^ cells) that were treated or not with rzMmp25b or rhMMP25. **k**, Coomassie blue staining of human placental collagen IV exposed or not to rhMMP25. The solid arrowheads indicate parental fragments (black) and rhMMP25 cleavage products (red) analysed by MS. The open arrowheads indicate additional differences. **l**, Collagen IV and its most C-terminal non-tryptic peptide identified in rhMMP25-treated samples. NC, non-collagenous. **m**, Anti-GFP western blot analysis of recombinant GST–GFP fusion proteins (black arrowheads) and cleavage products (white arrowheads). GST and GFP are linked by a PreScission recognition site and the presumptive cleavage site of MMP25 in COL4A1–6 (a1 to a6). C^−^, GST alone. The diagram was created using BioRender. For **h** and **i**, data are median ± interquartile range. *P* values were calculated using nonparametric Kruskal–Wallis tests. Scale bars, 500 nm (**c** (bottom)), 200 μm (**f** (left)), 100 μm (**a**, **b**, **d** and **e** (top) and **g**), 50 μm (**f** (right)), 20 μm (**a**, **b**, **d** and **e** (bottom)) and 2 μm (**c** (top)). Source Data

**Extended Data Fig. 9:**
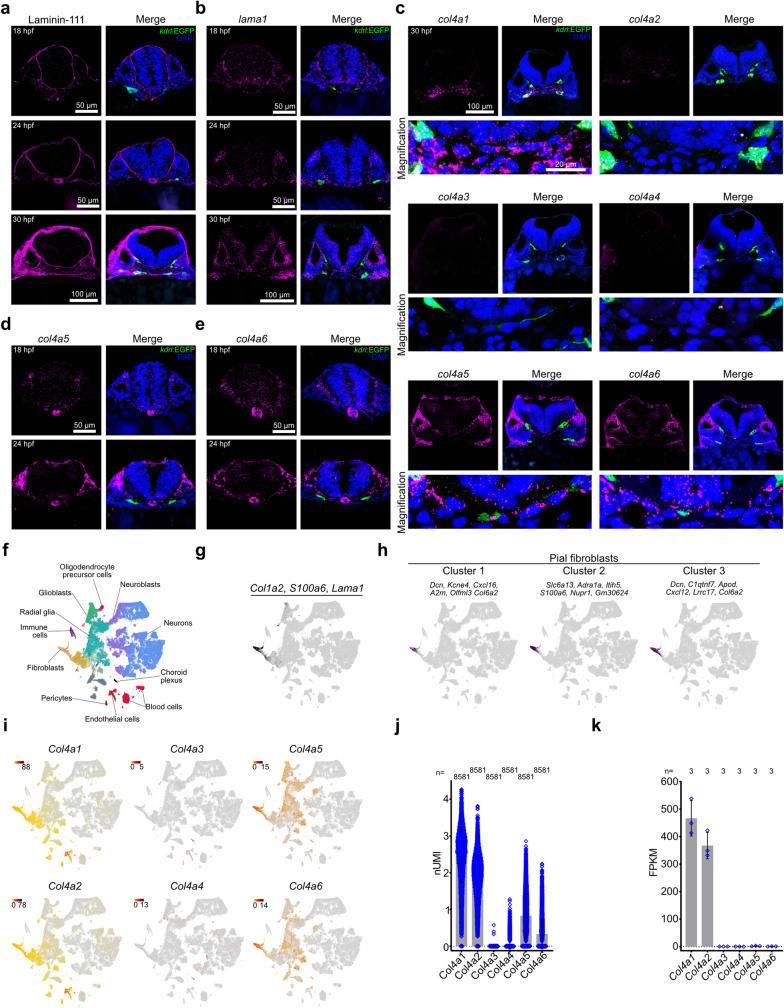
Collagen IV isoform expression in zebrafish and mouse meningeal fibroblasts. **a**, Immunostaining for laminin-111 on transverse hindbrain sections of 18, 24, and 30 hpf WT *Tg(kdrl:EGFP)* embryos, counterstained with DAPI. **b**, Combined fluorescent in situ hybridization for *lama1* and immunostaining for EGFP of 18, 24, and 30 hpf WT *Tg(kdrl:EGFP)* transverse hindbrain sections, counterstained with DAPI. In **a** and **b**, the 30 hpf images are the same as in Figs. 3a and 3b, to facilitate the direct comparison with the earlier stages. **c**, Combined fluorescent in situ hybridization for *col4a1-6* and immunostaining for EGFP of 30 hpf WT *Tg(kdrl:EGFP)* transverse hindbrain sections, counterstained with DAPI. The *col4a5* and *col4a6* images are the same as in Figs. 3d and 3e, respectively, to facilitate the comparisons with the other *col4a* isoforms. **d**,**e**, Combined fluorescent in situ hybridization for *col4a5* (**d**) or *col4a6* (**e**) and immunostaining for EGFP of 18 and 24 hpf WT *Tg(kdrl:EGFP)* transverse hindbrain sections, counterstained with DAPI. **f**, Cluster annotation of a whole brain scRNA-Seq analysis during embryonic stages, adapted from La Manno et al.^40^. **g**, Cell populations co-expressing the pan-fibroblast marker *Col1a2* and the meningeal specific fibroblast markers *S100a6* and *Lama1*. **h**, Pial fibroblast clusters and their marker genes as determined by unsupervised clustering. **i**, Expression profiles of *Col4a1-6* isoforms. **j**, Normalized expression values for *Col4a1-6* in mouse meningeal fibroblasts (*Col1a1*^*+*^) at E14.5^42^. **k**, Normalized counts for *Col4a1-6* transcripts in ECs (CD31^+^; CD45^−^) at E15.5^44^. In **j** and **k**, Data represent mean ± standard deviation. n = number of individual meningeal fibroblasts (j) or brain endothelia (k) analysed by transcriptomics.

To identify the physiologically relevant Mmp25 substrate within the poorly characterized pBM, we transcriptionally characterized the meningeal fibroblasts, the main suppliers of pBM proteins^41,42^. We first analysed the anatomical relationships between the zebrafish pBM (anti-laminin-111), the meningeal fibroblasts (fluorescence in situ hybridization (FISH) analysis of *lama1*), a cell population that is to date uncharacterized in this organism, and the *kdrl:*EGFP^+^ ECs (Fig. 3a,b). The laminin-111-positive pBM was evident from 18 hpf onwards (Fig. 3a and Extended Data Fig. 9a), with the nearest *lama1* signal associated with a ventrolateral population of perineural cells (Fig. 3b and Extended Data Fig. 9b). At 18 hpf, these cells probably represent primary meninx cells, meningeal fibroblast precursors. Between 24 and 30 hpf, the *lama1*^*+*^ cells flattened out on the ventral surface of the hindbrain, with the equatorial plane of their oblong nuclei orienting parallel to the pBM (Fig. 3a,b). Transmission electron microscopy revealed a typical elongated fibroblast cell morphology (Fig. 3c). These cells resemble pial fibroblasts, although we prefer to use the broader meningeal fibroblast terminology, as the molecular diversity of the zebrafish meningeal cell populations remains to be investigated.

Using a candidate-based approach, we identified that zebrafish meningeal fibroblasts expressed *col4a5* and *col4a6*, specific chains of type IV collagen, in a spatiotemporal pattern very similar to *lama1* (Fig. 3d,e and Extended Data Fig. 9c–e). Exploration of published mouse transcriptomes confirmed the expression of *Col4a5* and *Col4a6* by embryonic and adult pial fibroblasts^40,42,43^ (Extended Data Fig. 9f–j), together with *Col4a1* and *Col4a2*, common components of EC basement membranes^40,42–44^ (Extended Data Fig. 9k). Immunostaining analysis confirmed the presence of type IV collagen in the laminin-111-positive E10.5 mouse pBM (Fig. 3f).

**Extended Data Fig. 10:**
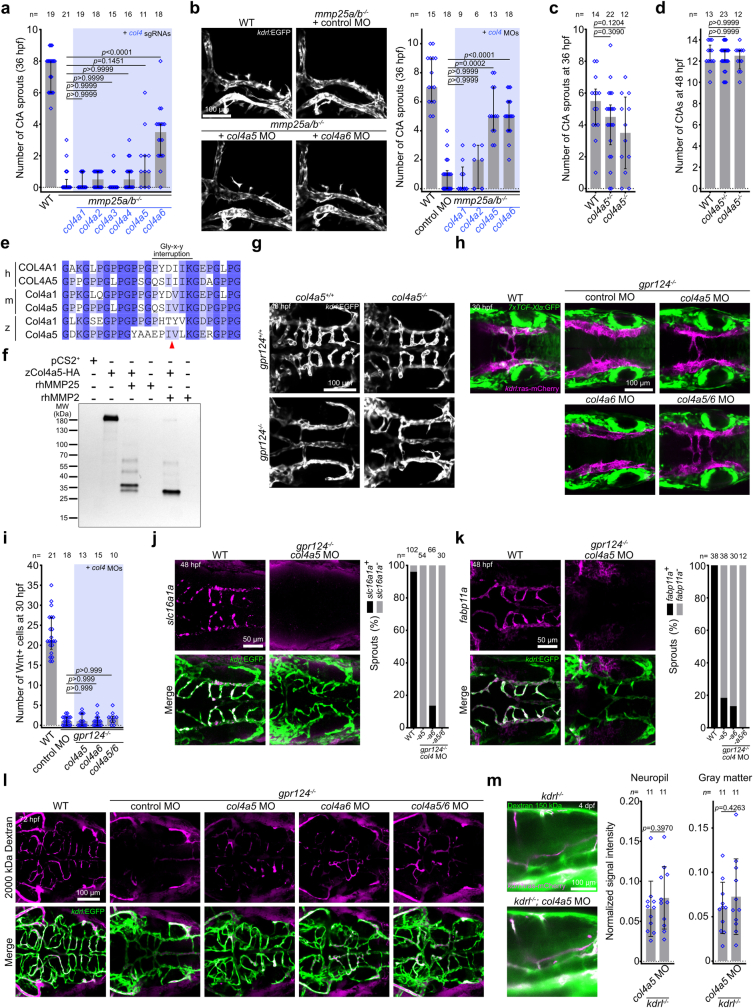
Genetic interaction between *col4a5/6* and *mmp25* or *gpr124* during brain angiogenesis. **a**, Quantification of hindbrain CtA sprouts in 36 hpf *mmp25a/b* mutant embryos injected at the one-cell stage with 150 pg of *zCas9* mRNA and 60 pg of the indicated *col4*-targeting sgRNAs. **b**, Lateral views and quantification of hindbrain CtA sprouts in 36 hpf *Tg(kdrl:EGFP) mmp25* mutant embryos injected at the one-cell stage with a control or *col4*-targeting morpholinos. **c,d**, Quantification of hindbrain CtA sprouts in WT, *col4a5*^*+/−*^ or *col4a5*^*−/−*^ embryos at 36 (**c**) and 48 hpf (**d**). In **a-d**, n ≥ 6 embryos from 3 independent experiments. **e**, Amino acid sequence alignment (Jalview v2.11.3.2) of the Mmp25 cleavage site (red arrowhead) within human (h), mouse (m), and zebrafish (z) Col4a1 and Col4a5. **f**, Anti-HA western blot of zCol4a5-HA-containing HEK293T extracts (or control pCS2^+^) treated or not with rhMMP25 or rhMMP2. **g**, Dorsal views of WT, *gpr124* and *col4a5* single or double mutant *Tg(kdrl:EGFP)* embryos at 48 hpf. **h,i**, Dorsal views (**h**) and quantification (**i**) of *7xTCF:*GFP^+^ PHBC ECs in 30 hpf *Tg(7xTCF-Xla.Siam:GFP);(kdrl:ras-mCherry)* embryos injected with control, *col4a5* and/or *col4a6* MOs. n ≥ 10 embryos from 5 independent experiments. In **a-d**,**i**, data represent median ± interquartile range. p-values were determined using the non-parametric Kruskal-Wallis test. **j**,**k**, Combined fluorescent WISH for *slc16a1a* (**j**) or *fabp11a* (**k**) and immunostaining for EGFP in *Tg(kdrl:EGFP)* embryos injected at the one-cell stage with *col4a5* and/or *col4a6* MOs. On the right, proportion of *slc16a1a* or *fabp11a*-positive 48 hpf CtAs. In **j** and **k**, n = number of CtAs from 3 independent experiments. **l**, 2000 kDa dextran-rhodamine angiography in 72 hpf WT and *gpr124*^*−/−*^ *Tg(kdrl:EGFP)* larvae injected with control or *col4a5/6* morpholinos. **m**, 1 h before imaging, FITC-labelled 150 kDa dextran was injected intracardially into *kdrl*^*−/−*^ 4 dpf *(kdrl:ras-mCherry)* larvae injected or not with a *col4a5*-targeting MO. Tracer accumulation was normalized to the intra-luminal fluorescence of the lateral dorsal aorta. n = 11 embryos from 3 independent experiments. Data represent mean ± SD. p-values were determined using the parametric two-tailed Student’s t-test. Source Data

We next inactivated the different zebrafish *col4* chains by MOs and somatic CRISPR–Cas9 mutagenesis in *mmp25a/b*^*−/−*^ embryos. Selectively targeting *col4a5* or *col4a6* rescued the cerebrovascular defects partially at 36 hpf and nearly completely at 48 hpf (Fig. 3g,h and Extended Data Figs. 8a,b and 10a,b). This finding was confirmed through Mendelian genetics by crossing the *mmp25-*mutant alleles to the *dragnet* allele^45^, harbouring a premature stop codon in *col4a5* (Fig. 3i). The inactivation of *col4a5* therefore alleviates the need for Mmp25 in brain angiogenesis. Notably, brain angiogenesis in *dragnet* mutants proceeded as in WT siblings, despite an initial non-significant reduction in CtA sprouts at 36 hpf (Extended Data Fig. 10c,d).

Recombinant Mmp25 catalytic domains cleaved C-terminally HA-tagged zebrafish Col4a5 (zCol4a5–HA) in HEK293T lysates, yielding at least four distinct fragments including a major ~30 kDa band (Fig. 3j). This suggests that the central triple-helix domain of Col4a5 contains an Mmp25-cleavage site, thereby providing a link between the proteolytic activity of Mmp25 and its function in EC migration across the pBM.

To identify the cleavage site, we used a two-step strategy as purified Col4a5–Col4a6 heterotrimer is not commercially available. In step one, we exposed purified human placental collagen IV, which is mostly composed of COL4A1/A2, to rhMMP25. Untreated samples ran as multiple bands, probably a consequence of the harsh pepsin-based placental extraction procedure. rhMMP25 cleaved the main bands (Fig. 3k). The two larger parental bands and their presumptive cleavage products were analysed using mass spectrometry (MS), which revealed a semi-specific (K)GLPGPPGPPGPYD peptide in rhMMP25-treated collagen IV, containing a C-terminal non-tryptic aspartate (Fig. 3l). By contrast, the tryptic (K)GLPGPPGPPGPYDIIK fragment ending with a lysine residue was found in untreated collagen IV samples. MMPs often cleave proteins upstream of two small hydrophobic residues, consistent with the observed (Asp)-Ile-Ile cleavage site in human COL4A1, and the corresponding residues in Col4a1 and Col4a5 across vertebrates (Extended Data Fig. 10e). The peptide mapped to the last short non-collagenous region within the central helical part of the α-chain, upstream of the C-terminal globular domain, essential for collagen IV sheet formation (Fig. 3l). In step two, to confirm that Mmp25 can process Col4a5 or Col4a6 at this site, we expressed tripartite fusion constructs in *Escherichia coli*, consisting of an N-terminal glutathione *S*-transferase, a C-terminal GFP and a central linker containing the presumptive cleavage site of Col4 chains in tandem with the recognition site of the human rhinovirus-derived PreScission enzyme (used as a positive control). Recombinant hMMP25 processed all Col4-based fusions, while rhMMP2 did not, revealing qualitative differences in collagen IV cleavage by Mmp2 and Mmp25 (Fig. 3m). Such differences were confirmed on full-length zebrafish Col4a5, which was cleaved by both enzymes, but yielded proteolytic fragments of different sizes (Extended Data Fig. 10f).

## Uncoupled angiogenesis and BBB formation

**Fig. 4:**
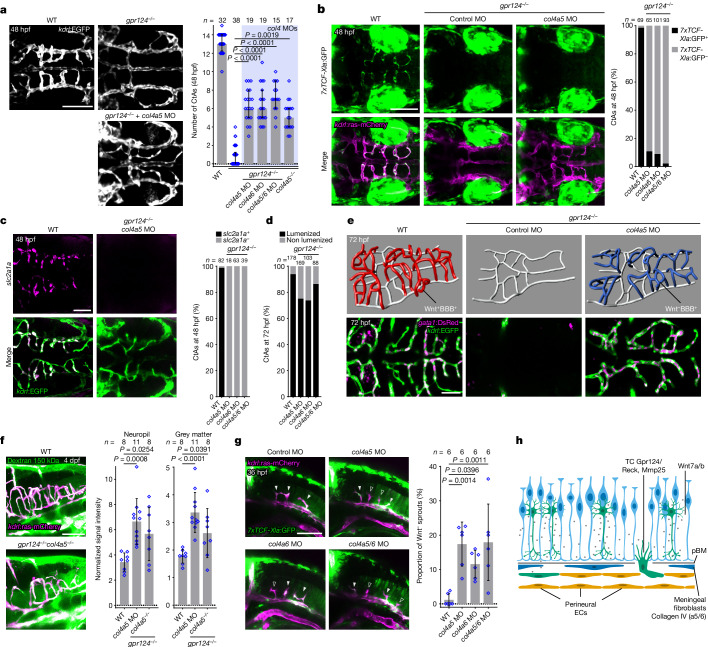
Col4a5/6 inactivation unlocks the quality control of brain angiogenesis by Wnt–β-catenin signalling. **a**, Hindbrain CtAs in 48 hpf *Tg(kdrl:EGFP)* embryos (*n* ≥ 15 embryos from ≥3 independent experiments). Data are median ± interquartile range. *P* values were calculated using nonparametric Kruskal–Wallis tests. **b**, The proportion of *7xTCF-Xla:*GFP^+^ CtAs in *Tg(7xTCF-Xla.Siam:GFP);(kdrl:ras-mCherry)* 48 hpf embryos. **c**, The proportion of *slc2a1a*-positive CtAs was analysed using fluorescent *slc2a1a* WISH and anti-EGFP immunostaining in *Tg(kdrl:EGFP)* embryos. **d**, The percentage of lumenized CtAs in 72 hpf larvae. For **b**–**d**, *n* = total number of CtAs from 3 (**b** and **c**) or 5 (**d**) independent experiments. **e**, Wire diagrams (top) and dorsal views (bottom) of 72 hpf *Tg(gata1:DsRed);(kdrl:EGFP)* larvae. **f**, FITC 150 kDa dextran fluorescence intensity 1 h after intracardial injection in larvae at 4 days post-fertilization (dpf). *n* ≥ 8 larvae from ≥4 independent experiments. **g**, The proportion of *7xTCF-Xla:*GFP^–^ CtA TCs in *Tg(7xTCF-Xla.Siam:GFP);(kdrl:ras-mCherry)* embryos. The solid and open arrowheads label *7xTCF*-*Xla*:GFP^+^ and *7xTCF*-*Xla*:GFP^–^ sprouts, respectively. *n* = 6 independent experiments with ≥6 embryos each. For **f** and **g**, data are mean ± s.d. *P* values were calculated using parametric one-way ANOVA. **h**, Model for brain-specific angiogenesis. The diagram was created using BioRender. Scale bars, 100 μm (**a**, **b**, **f** and **g**) and 50 μm (**c** and **e**). Source Data

Thus far, we have identified that Wnt–β-catenin signalling, by regulating *mmp25* expression, enables the migration of TCs across the Col4a5/6-positive pBM. As Wnt–β-catenin also controls BBB development, this mechanism ensures brain perfusion by vessels led by TCs of adequate properties. The coupling between brain angiogenesis and BBB formation therefore appears to rely on the integrity of the pBM. If true, this model implies that, after impairment of the pBM, Wnt–β-catenin would at least in part become dispensable for brain angiogenesis. Consistent with this prediction, brain angiogenesis was partially restored in *gpr124* mutants after *col4a5* and/or *col4a6* inactivation (Fig. 4a and Extended Data Fig. 10g). Under these conditions, brain vessels remained Wnt–β-catenin negative (Fig. 4b and Extended Data Fig. 10h,i) and, accordingly, did not express BBB markers such as *slc2a1a*, *slc16a1a* or *fabp11a* (Fig. 4c and Extended Data Fig. 10j,k). The vessels lumenized properly (Fig. 4d and Extended Data Fig. 10l), conducted *gata1*^*+*^ red blood cells (Fig. 4e), assembled vasculatures notably similar to WT networks (Fig. 4e), but remained leaky to intracardially injected tracers (Fig. 4f). Tracer accumulation did not result from leakage through the impaired pBM, as it was not observed in the absence of cerebral vessels (Extended Data Fig. 10m). Together, these observations reveal that, after pBM impairment, the organotypic requirement of Wnt signalling for brain vascularization is lost, resulting in properly patterned, yet BBB-defective, cerebrovasculatures. A last prediction from the pBM-mediated quality control on TCs is that not only *gpr124*^*−/−*^ cells would gain undue access to the CNS after pBM impairment, but also the naturally occurring Wnt-negative TCs populating the PHBC (Fig. 1d and Extended Data Fig. 2a,b). Consistent with this prediction, Wnt-negative TCs invaded the hindbrain of *col4a5* and/or *col4a6* morphants (Fig. 4g). The pBM therefore appears to control the genetic competence of the perineural vessels to differentiate into a neuroprotective BBB, by controlling the pioneering TCs. Either vessels are Wnt competent, displaying the right cohort of cell surface receptors, and Mmp25-expressing TCs will guide nascent sprouts into the CNS, or they are not, and they are excluded.

## Discussion

Vascular expansion through angiogenesis is a multicellular migration process that requires the coordinated behaviours of differentially fated TCs and SCs. TCs invariably guide the nascent angiogenic sprouts and therefore display common morphological and molecular adaptations required for tissue exploration. Beyond this apparent uniformity, TCs, in their task to pervade each vertebrate organ, must navigate through extracellular spaces of varying composition likely imposing local constraints on TC function. Here we provide an important example of such TC mechanistic angiodiversity by identifying Mmp25 as a brain-specific TC angiogenic effector.

An alluring aspect of this brain-specific TC machinery is that Wnt–β-catenin signalling, besides conferring organ invasive competence, also instructs endothelial tissue-specific adaptations. This constitutes an elegant quality-control mechanism ensuring that only BBB-differentiating TCs can guide vessels into the brain. However, neuroprotection implies a uniform activity in Wnt–β-catenin among all CNS ECs, not only TCs. How and if a quality-control mechanism operates at the level of naturally occurring Wnt-negative cells other than TCs remains to be investigated. Notably, studies in the postnatal mouse retina detected such a potential process, in which Wnt-signalling-deficient *Frizzled4*^*−/−*^ ECs were gradually eliminated from genetically mosaic vessels^46^.

At the centre of the brain-specific angiogenic program lies the pial (or glia limitans) BM (Fig. 4h). This work extends the function of this critical interface between the brain and the periphery. To date, it has been implicated in proper cortical layering, by acting as a scaffold for radial glia endfeet and avoiding the inside-out overmigration of neurons and glia into the meninges, a condition known in humans as cobblestone lissencephaly^34,36,37,47^. We show that the pBM also constitutes an important gatekeeper of the brain by hindering the uncontrolled ingression of peripheral cells such as leaky ECs. In doing so, here we reveal a functional connection between two important brain barriers, that is, the BBB and the meningeal barrier to which the pia and its basement membrane belong. This study also illustrates the increasingly recognized importance of fibroblasts in brain barrier function, both within and at the surface of the brain^42,43,48^. Besides its angiogenic role identified in this study, Mmp25 is better known to be expressed by leukocytes, particularly neutrophils^19,23^. Whether Mmp25 facilitates immune cell entry across the glia limitans in pathological conditions like infections, trauma and haemorrhage seems worth pursuing in light of the results presented here.

Together, we reveal a mechanism by which endothelial TCs gain brain-specific invasive competence, thereby supporting the existence of specialized TC angiogenic mechanisms in distinct organs, including in the bone, liver, retina and solid tumours, where diverse TC morphologies or gene signatures are increasingly reported^49,50^. Given the essential role of TCs in guiding new vessels, such organotypic TC functional heterogeneity holds promise for tissue-specific pharmacological control of angiogenesis, at a level of specificity that is unachievable using the current anti-angiogenic strategies.

## Methods

### Zebrafish strains and husbandry

Zebrafish (*Danio rerio*) were maintained at 28 °C under a 14 h–10 h light–dark cycle and raised under standard conditions in a certified animal facility (LA1500474) in accordance with European and national ethical and animal welfare guidelines. All of the animal procedures were approved by the corresponding ethical committee (Commission d’Ethique et du Bien Être Animal (CEBEA), Université libre de Bruxelles, protocol approval numbers: CEBEA-IBMM-2016:65 and CEBEA-07 GOS IBMM). Zebrafish staging was performed as described previously^51^. The following published transgenic and mutant lines have been used in this study: *Tg(kdrl:EGFP)*^*s843*^ (ref. ^52^)*, Tg(kdrl:ras-mCherry)*^*s896*^ (ref. ^53^), *Tg(7xTCF-Xla.Siam:GFP)*^*ia4*^ (ref. ^54^), *Tg(fli1a:Gal4FF)*^*ubs3*^ (ref. ^55^), *Tg(UAS:Kaede)*^*rk8*^ (ref. ^56^), *Tg(UAS:GCaMP7a)*^*zf415*^ (ref. ^57^), *Tg(gata1:DsRed)*^*sd2*^ (ref. ^58^), *gpr124*^*s984*^ (ref. ^11^), *wnt7aa*^*ulb2*^ (ref. ^17^), *reck*^*ulb3*^ (ref. ^59^), *kdrl*^*hu5088*^ (ref. ^60^) and *col4a5*^*s510*^ (ref. ^45^). The *mmp25a*^*ulb26*^ and *mmp25b*^*ulb27*^ alleles were generated in this study using CRISPR–Cas9 mutagenesis. All of the zebrafish experiments were performed on embryos and larvae younger than 5 days post-fertilization, before they became capable of independent feeding.

### Mice

Mice were housed at 20 °C under a 12 h–12 h light–dark cycle under standard conditions and were maintained in a certified animal facility (LA1500474) in accordance with European and national ethical and animal welfare guidelines. The relative ambient humidity level ranged from 45 to 65%. All animal procedures were approved by the corresponding ethical committee (Commission d’Ethique et du Bien Être Animal (CEBEA), Université libre de Bruxelles, protocol approval number: CEBEA-08 GOS IBMM). Mice were maintained on the C57BL/6J background and, for experiments, mice of both sexes were used. BAT-GAL reporter (B6.Cg-Tg(BAT-LacZ)3Picc/J) mice^61^ and *Mmp25-*knockout mice^23^ were provided by S. Piccolo and C. López-Otín, respectively. Vascular networks were quantified as the number of CNS-invading sprouts in the E10.5 midbrain and forebrain in five consecutive 60 μm sections, and as the organ surface-normalized vascular density (length or surface, depending on the vascular morphologies) in 60 μm sections of E10.5 forelimbs and E12.5 intestine, stomach, liver and lung.

### CRISPR–Cas9-mediated gene disruption in zebrafish

Germline zebrafish *mmp25a*^*ulb26*^ and *mmp25b*^*ulb27*^ alleles were generated using CRISPR–Cas9 as described previously^62^. Target sites were selected using CRISPOR (v.5.01)^63^. The following primers were annealed and cloned into the pT7-gRNA vector (Addgene, 46759): 5′-TAGGGGCAATGCCCTGCGAGTG-3′ and 5′-AAACCACTCGCAGGGCATTGCC-3′ for *mmp25a*; 5′-TAGGGGACAGCTACAGAGCAAAGA-3′ and 5′-AAACTCTTTGCTCTGTAGCTGTCC-3′ for *mmp25b*. sgRNAs were synthesized by in vitro transcription (HiScribe T7 Quick High Yield RNA Synthesis Kit; New England Biolabs) from BamHI-linearized pT7-gRNA vectors. *Mmp25a* was targeted in exon 4 (catalytic domain) and *mmp25b* was targeted in exon 2 (pro-domain). Synthetic capped *zCas9* mRNA was transcribed from the XbaI-linearized pT3TS-nls-zCas9-nls vector (Addgene, 46757) using the mMESSAGE mMACHINE T3 Kit (Ambion). Co-injection of the sgRNAs (30 pg each) and *nls-zCas9-nls* mRNA (150 pg) was performed at the one-cell stage.

For somatic gene disruptions, two sgRNAs targeting the same exon were synthesized using the following primer pairs: *mmp2* sgRNA1: 5′-TAGGGGGAACTTTATGATGGGTG-3′ and 5′-AAACCACCCATCATAAAGTTCCC-3′; *mmp2* sgRNA2: 5′-TAGGGGAACTTTATGATGGGTGA-3′ and 5′-AAACTCACCCATCATAAAGTTCC-3′; *mmp14b* sgRNA1: 5′-TAGGCCAGTCCATTTGATGGAGA-3′ and 5′-AAACTCTCCATCAAATGGACTGG-3′; *mmp14b* sgRNA2: 5′-TAGGATTCCCTGGGAAGTAAGCAT-3′ and 5′-AAACATGCTTACTTCCCAGGGAAT-3′; *mmp25a* sgRNA1: 5′-TAGGGGCAATGCCCTGCGAGTG-3′ and 5′-AAACCACTCGCAGGGCATTGCC-3′; *mmp25a* sgRNA2: 5′-TAGGGTCTGGTGAGGCTTATTTT-3′ and 5′-AAACAAAATAAGCCTCACCAGAC-3′; *mmp25b* sgRNA1: 5′-TAGGTAGGACTGGTTGAGCCGGTA-3′ and 5′-AAACTACCGGCTCAACCAGTCCTA-3′; *mmp25b* sgRNA2: 5′-TAGGAGGAGGCAGATATCCATAC-3′ and 5′-AAACGTATGGATATCTGCCTCCT-3′; *lama1* sgRNA1: 5’-TAGGGAACGGCCGTCAGTTCCACT-3′ and 5′-AAACAGTGGAACTGACGGCCGTTC-3′; *lama1* sgRNA2: 5′-TAGGCGGACTCTGCCACCACAGGT-3′ and 5′-AAACACCTGTGGTGGCAGAGTCCG-3′; *lama1* sgRNA1-scrambled: 5′-TAGGGAACGGCCGTCAGTTACCTC-3′ and 5′-AAACGAGGTAACTGACGGCCGTTC-3′; *lama1* sgRNA2-scrambled: 5′-TAGGCGGACTCTGCCACCGATGAC-3′ and 5′-AAACGTCATCGGTGGCAGAGTCCG-3′; *lama2* sgRNA1: 5′-TAGGCGCAGACAGGCTCCGGTCAG-3′ and 5′-AAACCTGACCGGAGCCTGTCTGCG-3′; *lama2* sgRNA2: 5′-TAGGTCAGCGGGTCACAGCTCAG-3′ and 5′-AAACCTGAGCTGTGACCCGCTGA-3′. *lama2* sgRNA1-scrambled: 5′-TAGGCGCAGACAGGCTCCACGGGT-3′ and 5′-AAACACCCGTGGAGCCTGTCTGCG-3′; *lama2* sgRNA2-scrambled: 5′-TAGGTCAGCGGGTCACATGCAGC-3′ and 5′-AAACGCTGCATGTGACCCGCTGA-3′; *col4a1* sgRNA1: 5′-TAGGATAGGTCCTGGCGGTCCGGG-3′ and 5′-AAACCCCGGACCGCCAGGACCTAT-3′; *col4a1* sgRNA2: 5′-TAGGCAGGTCCCAAAGGAACTGAT-3′ and 5′-AAACATCAGTTCCTTTGGGACCTG-3′; *col4a2* sgRNA1: 5′-TAGGTGGCAGTCCCGGATCTCCAG-3′ and 5′-AAACCTGGAGATCCGGGACTGCCA-3′; *col4a2* sgRNA2: 5′-TAGGAGGTTTGGATGGAGCTTCAG-3′ and 5′-AAACCTGAAGCTCCATCCAAACCT-3′; *col4a3* sgRNA1: 5′-TAGGAAGGTTGTGCTGGGGTTCA-3′ and 5′-AAACTGAACCCCAGCACAACCTT-3′; *col4a3* sgRNA2: 5′-TAGGAAGGATTCCCAGGATTGTGT-3′ and 5′-AAACACACAATCCTGGGAATCCTT-3′; *col4a4* sgRNA1: 5′-TAGGTGGGTCGACAGGGCCCCCAG-3′ and 5′-AAACCTGGGGGCCCTGTCGACCCA-3′; *col4a4* sgRNA2: 5′-TAGGAGAACCTTGGGGCCCCTGG-3′ and 5′-AAACCCAGGGGCCCCAAGGTTCT-3′; *col4a5* sgRNA1: 5′-TAGGCCTGGGAAACCTGGAACACC-3′ and 5′-AAACGGTGTTCCAGGTTTCCCAGG-3′; *col4a5* sgRNA2: 5′-TAGGCCGGGTTTAAAGGGTCAGCC-3′ and 5′-AAACGGCTGACCCTTTAAACCCGG-3′; *col4a6* sgRNA1: 5′-TAGGCTTGGACCAGTGGGCAGCGG-3′ and 5′-AAACCCGCTGCCCACTGGTCCAAG-3′; *col4a6* sgRNA2: 5′-TAGGATGGGGGCCCGGGACCAGTT-3′ and 5′-AAACAACTGGTCCCGGGCCCCCAT-3′; *serpina1* sgRNA1: 5′-TAGGTGCTGCCTTGCTGGTAGCAA-3′ and 5′-AAACTTGCTACCAGCAAGGCAGCA-3′; *serpina1* sgRNA2: 5′-TAGGCTGGTAGCAACGGCCTGGG-3′ and 5′-AAACCCCAGGCCGTTGCTACCAG-3′.

The efficiency of somatic gene disruption was scored by high-resolution melt analysis (HRMA) using the Illumina Eco real-Time PCR system, and further characterized using Illumina amplicon deep sequencing (Azenta Life Sciences).

### Genotyping

Zebrafish *gpr124*^*s984*^, *wnt7aa*^*ulb2*^, *kdrl*^*hu5088*^, *reck*^*ulb3*^ and *col4a5*^*s510*^ and mouse *Mmp25* alleles were genotyped as described previously^11,17,23,45,59,60^. The *mmp25a*^*ulb26*^ and *mmp25b*^*ulb27*^ alleles were genotyped by high-resolution melt analysis (Eco Illumina real-time PCR system) using the following primers: 5′-TTTCCACCTCCCTCAGTGTC-3′ and 5′-GTGGAAACGCAGAGGTGTGT-3′ for *mmp25a*; 5′-CGCACAGGACAGCTACAGAG-3′ and 5′-CTGCATTTCTCTAATGGCTCTCTCG-3′ for *mmp25b*.

### MO, RNA and DNA microinjection in zebrafish

MOs targeting *gpr124* (4 ng; splice blocking; ACTGATATTGATTTAACTCACCACA)^11^, *reck* (0.4 ng; splice blocking; CAGGTAGCAGCCGTCACTCACTCTC)^64^, *wnt7aa* (4 ng; splice blocking; TTCCATTTGACCCTACTTACCCAAT)^17^, *lama1* (0.5 ng; translation blocking; ATCTCCATCATCGCTCAAACTAAAG), *lama2* (1 ng; translation blocking GCCACTAAACTCCGCGTGTCCATGT), *lama4* (0.5 ng; translation blocking; GCCATGATTCCCCCTGCAACAACTT), *lama5* (0.25 ng; translation blocking; CTCGTCCTGATGGTCCCCTCGCCAT)^65^, *lamb1a* (0.125 ng; translation blocking; TATTTCCAGTTTCTTTCTTCAGCGG), *lamc1* (0.125 ng; translation blocking; TGTGCCTTTTGCTATTGCGACCTC)^66^, *col4a1* (1 ng; translation blocking; ACACATGGAAGCCGCATCTTCACAC)^67^, *col4a2* (2 ng; translation blocking; TTCTCACCCTCCATGCGAGCCTAAA), *col4a5* (2 ng; translation blocking; ATGTTCCTCTGTTAAGCTAACTGCA), *col4a6* (2 ng; translation blocking; AGGTAAAGTAGGCTATCCTCCTCGT) were obtained from Gene Tools and were injected at the zygotic stage at the indicated doses. Injection of a standard control MO (CCTCTTACCTCAGTTACAATTTATA, up to 8 ng) did not affect the brain vasculature.

Transgenic mosaic endothelial overexpression was achieved by co-injecting at the one-cell stage 25 pg of *Tol2* transposase mRNA and 25 pg of the pTol2-fli1a:kdrl-2A-nls-mtagBFP2, pTol2-fli1a:mmp25b-2A-tagRFP, pTol2-fli1a:mmp25b^ΔZn2+-BD^-2A-tagRFP or pTol2-fli1a:mmp25b^Zn2+-BDH237A,H241A,H247A^-2A-tagRFP constructs^68^.

Capped mRNAs were transcribed in vitro from NotI-linearized pCS2^+^ constructs, using the mMessage mMachine SP6 Kit (Thermo Fisher Scientific) and injected at the one-cell stage at a dose of 200 pg. The fragment encoding the Zn^2+^-binding domain (Zn^2+^-BD; His237–His247) was deleted in the ΔZn^2+^-BD *mmp25b* variant. Three histidines, essential for Zn^2+^ chelation, were substituted by alanines in the Zn^2+^-BD^H237A, H241A, H247A^ variant, abbreviated as Zn^2+^-BD^H-A^. In the Pro^−^ *mmp2* mRNA variant, the sequences encoding the prodomain (Ala30–Val107) were deleted. The sequences corresponding to the GPI-anchoring site of Mmp25b (Ser658–Gln697) were fused 3′ to the *mmp2* ORF in the GPI^+^ *mmp2* variant.

### Transplantations

Host *Tg(kdrl:ras-mCherry)*^*s896*^ and donor *Tg(kdrl:EGFP)*^*s843*^ embryos were dechorionated with pronase (Millipore, 53702; 1 mg ml^−1^) during 5 min at 28 °C in 1/3 Ringer solution, supplemented with penicillin (50 U ml^−1^) and streptomycin (50 µg ml^−1^). The embryos were subsequently incubated on agarose-coated dishes in the same medium. At the mid-blastula stage, 20 to 50 donor cells were transplanted into the blastoderm margin of stage-matched host embryos. After transplantation, embryos were incubated until the indicated stages. After assessing the contribution of EGFP^+^ transplanted cells using the Leica M165 stereomicroscope, mosaic vessels were recorded using time-lapse confocal microscopy. The contribution of cells of a defined genotype to the TC position was calculated as the fraction of the total number of mosaic vessels (CtAs or ISVs). The contribution to TC position in intraneural secondary branches was scored as the fraction of the stalk cell genotype in the initial brain-invading CtA.

### Immunofluorescence and in situ hybridization

Zebrafish and mouse embryos were fixed in 4% paraformaldehyde (PFA) in PBS. For sections, embryos were washed in PBS and equilibrated in 30% sucrose in PBS (w/v) overnight at 4 °C. The embryos were then mounted in 7.5% gelatin (w/v), 15% sucrose (w/v) in PBS and stored at −80 °C. Zebrafish and mouse embryos were cut into 20 and 60 µm frozen sections, respectively, using the Leica CM1850 Cryostat (Leica) at −30 °C.

For immunofluorescence staining, the sections were washed three times with PBS Triton X-100 (0.4%; PBST) for 5 min, blocked using blocking buffer (PBST, 5% goat serum) for 1 h and then incubated with primary antibodies in blocking buffer solution overnight at 4 °C. After three washing steps in PBST for 5 min, the sections were exposed to secondary antibodies diluted in blocking buffer containing 0.001% DAPI overnight at 4 °C. After three washing steps in PBST for 5 min, the sections were mounted in DAKO fluorescence mounting medium (Agilent, S3023). The following primary antibodies and lectin were used: rabbit anti-laminin-111 (Merck, L9393, 1:250, used for zebrafish immunostaining, polyclonal immunization with an Engelbreth–Holm–Swarm mouse sarcoma extract), rat anti-laminin-111 (R&D systems, MAB4656, 1:250, used for mouse immunostainings, monoclonal reactivity towards LAMA1/B1), chicken anti-GFP (Aves Labs, GFP-1020, 1:200), rabbit anti-collagen type IV (Sigma-Aldrich, AB756P, 1:300), chicken anti-β-galactosidase (Abcam, ab9361, 1:300), anti-Erg1-Alexa Fluor (AF) 647 conjugate (Abcam, ab196149, 1:250) and isolectin B4-AF594 conjugate (Thermo Fischer Scientific, I21413, 1:200). The following secondary antibodies were used: goat anti-chicken AF488 (Thermo Fischer Scientific, A11039, 1:500), goat anti-rabbit AF594 (Thermo Fischer Scientific, A11012, 1:500), and donkey anti-rat AF647 (Thermo Fischer Scientific, A48272, 1:500).

For in situ hybridization, digoxigenin (DIG)-labelled antisense riboprobes were produced by in vitro transcription using the DIG RNA labelling kit and SP6 RNA polymerase (Roche). The templates were amplified from 48 hpf WT embryo cDNA, and cloned into NcoI/SacI-digested pGEMT using the following primers: *kdrl*: 5′-GCATGCTCCCGGCCGCCATGGTGGCAGGATTCACTTTGAGTGG-3′ and 5′-CATCCAACGCGTTGGGAGCTCTAGTGTAGGGCTCAATCCGCAG-3′; *mmp25b*: 5′-ATGAGTTTCTCAGGATATCTTGGTCTGG-3′ and 5′-TTATTGCGAGTTGAAGCCAATATGAAGC-3′; *mmp14b:* 5′-GCATGCTCCCGGCCGCCATGGTGGATGCAGCTCTTCTCTACACG-3′ and 5′-CATCCAACGCGTTGGGAGCTCCATGAGGCTGCTGGAAATGTGC-3′; *mmp2*: 5′-GCATGCTCCCGGCCGCCATGGTGCTCACACAGACAAAGAAGTGG-3′ and 5′-CATCCAACGCGTTGGGAGCTCTTTCCTGACATCAGCCGTCC-3′; *mmp9*: 5′-GCATGCTCCCGGCCGCCATGGCAAATCTGTGTTCGTGACGTTTCC-3′ and 5′-CATCCAACGCGTTGGGAGCTCCTCCTTGATTTGGCAGGCATCG-3′; *lama1*: 5′-GCATGCTCCCGGCCGCCATGGGTCACAACAAAGCCGACGACTG-3′ and 5′-CATCCAACGCGTTGGGAGCTCTGAGCGTTCCCTCAGCGCTGT-3′; *col4a1*: 5′-GCATGCTCCCGGCCGCCATGGGGTTCTAAGGGTGAAGGAGGTG-3′ and 5′-CATCCAACGCGTTGGGAGCTCCCCTCTTCATGCACACTTGAC-3′; *col4a2*: 5′-GCATGCTCCCGGCCGCCATGGCCTAAAGGAGATACCGGACCC-3′ and 5′-CATCCAACGCGTTGGGAGCTCCTACAGGTTCTTCATGCACAC-3′; *col4a3*: 5′-GCATGCTCCCGGCCGCCATGGGGACAAAAAGGACAGTGTGGTC-3′ and 5′-CATCCAACGCGTTGGGAGCTCGCAAGGTCACCTTGAGGCTGTTG-3′, *col4a4*: 5′-GCATGCTCCCGGCCGCCATGGCTGGGTCCCAGTGGTGCAAAAG-3′ and 5′-CATCCAACGCGTTGGGAGCTCCATTGGTTGGGGTCATTCATC-3′; *col4a5*: 5′-GCATGCTCCCGGCCGCCATGGGGTTTTCCAGGATCTAAAGGAG-3′ and 5′-CATCCAACGCGTTGGGAGCTCCGTCCTCTTCATACACACCAC-3′; *col4a6*: 5′-GCATGCTCCCGGCCGCCATGGCGTCCAGGAATAATAGGACC-3′ and 5′-CATCCAACGCGTTGGGAGCTCCTACAAGATCTTCATGCAGAC-3′; *slc2a1a*: 5′-GCATGCTCCCGGCCGCCATGGCAACTTGGCATTGTCATTG-3′ and 5′-CATCCAACGCGTTGGGAGCTCGGCTGTGATCTCTTCAAACG-3′; *slc16a1a*: 5′-GCATGCTCCCGGCCGCCATGGATGCCTCCAGCAACAGGAGG-3′ and 5′-CATCCAACGCGTTGGGAGCTCCTATACGACTCCATCTGCCTCCTTTT-3′; *fabp11a*: 5′-GCATGCTCCCGGCCGCCATGGGATCAAATCTCAATTTACAGCTGTTG-3′ and 5′-CATCCAACGCGTTGGGAGCTCTTCAAAGCACCATAAAGACTGATAAT-3′. Whole-mount chromogenic in situ hybridizations were performed as previously described^69^ using anti-DIG-AP antibodies (Merck, 11093274910, 1:10,000). Combined immunostainings and FISH were performed as previously described^70^, using anti-DIG POD antibodies (Merck, 11207733910, 1:1,000) and the TSA Plus Cy3 detection kit (Akoya Biosciences, NEL744001KT).

### Photoconversion and FACS isolation of zebrafish brain ECs

Photoconversion of *Tg(fli1a:Gal4FF)*^*ubs3*^*;(UAS:Kaede)*^*rk8*^ PHBC or CtA ECs was performed using the Zeiss LSM710 confocal microscope (Carl Zeiss, objective lenses: Plan-Apochromat ×20/0.8 M27), as described previously^71^. In brief, anaesthetized embryos were mounted laterally in 1% low-melting-point agarose and the fluorescent Kaede protein was photoswitched by scanning the selected region of interest (ROI) using a 405 nm laser (five iterations of 50 s). After isolation from the agarose, the embryos were washed in Ca^2+^/Mg^2+^-free Hank’s Balanced Salt Solution (HBSS, Gibco) and dissociated at 28.5 °C for 30 min in TrypLE select (Thermo Fischer Scientific, 12563011). Dissociation was stopped by the addition of FBS and centrifugation. The cell pellet was resuspended in HBSS containing Ca^2+^/Mg^2+^ and 5% FBS, filtered and submitted for FACS analysis (BD Biosciences FACSAria III).

For scRNA-seq analyses, single photoswitched (red fluorescent) WT ECs were distributed in individual wells of 384-well plates containing 2.3 µl of Smart-seq2 lysis buffer (0.2% Triton X-100, 2 U µl^−1^ RNase inhibitor, 2 mM dNTP mix and 1 µM Smart-seq2 primer (5′-AAGCAGTGGTATCAACGCAGAGTACT30VN-3′). The plates were stored at −80 °C before mRNA-seq using the Smart-Seq2 protocol^72^ and analysis using the Seurat v4 toolkit in Rstudio (v.1.1.463)^73^. In brief, single-cell fastq files were demultiplexed by applying standard parameters of the Illumina pipeline (bcl2fastq v.2.19.0.316) using Nextera XT index kit v2 adapters. Mapping was performed to the zebrafish reference genome build GRCz11, with TopHat v.2.1.1 and Bowtie1 or Bowtie2 option. Adapter sequences were removed using Trim Galore v.0.4.4 before read mapping and doublets were removed using Samtools v.1.16.1 software. The generated BAM files containing the alignment results were sorted according to the mapping position, and raw read counts for each gene were calculated using the FeatureCounts function from the Subread package v.1.4.6-p5. For technical control, 92 ERCC RNAs were included in the lysis buffer and in the mapping.

For bulk RNA-seq analyses, *Tg(fli1a:Gal4FF)*^*ubs3*^*;(UAS:Kaede)*^*rk8*^ embryos were injected, or not, at the one-cell stage with *gpr124*, *reck* or *wnt7aa* MOs and PHBC ECs were isolated at 30 hpf as described above. Alternatively, embryos were treated with IWR-1 from 26 hpf onwards and CtA ECs were photoconverted and sorted at 36 hpf, as described above. Photoconverted PHBC ECs of 80 embryos were pooled and submitted for RNA extraction and RNA-seq, as previously described^71^. Transcriptomes were analysed and compared using DESeq2 (v.1.12)^74^.

### Light microscopy image acquisition and processing

All images were acquired using the Leica M165 stereomicroscope, the Zeiss LSM710 or the Zeiss LSM900 confocal microscope equipped with the Leica Application Suite (LAS) v.4.2 or ZEN Blue v.3.1 microscopy software. Image analysis was performed using ImageJ v.1.53c. Zebrafish embryos were imaged live or after fixation in 4% PFA in PBS overnight at 4 °C. Mouse embryos were fixed (4% PFA in PBS), and stained after sectioning. Live imaging of dechorionated zebrafish embryos was performed after embryo immobilization with a low dose of tricaine in low-melting-point agarose (1% in E3 zebrafish medium supplemented with *N*-phenylthiourea and tricaine) in a glass-bottom Petri dish (MatTek Corporation). Confocal time-lapse images were recorded at a stable temperature of 28.5 °C, using an incubation chamber. Ca^2+^-oscillations were recorded by time-lapse imaging of *Tg(fli1a:Gal4FF);(UAS:GCaMP7a)* embryos, taking a *z* stack every 5 s during the 30 min before CtA sprouting (31–31.5 hpf). Circular ROIs (<5 µm diameter) were centred on oscillating PHBC ECs. *F*/*F*_0_ was calculated to quantify changes in fluorescence, where *F*_0_ is the baseline fluorescence. Ca^2+^ spikes were identified as events of *F*/*F*_0_ ≥ 1.5.

For angiography, imaging was performed 1 h after injection of 1 nl of tetramethylrhodamine dextran 2,000,000 Da molecular mass (Thermo Fisher Scientific, D7139, 25 μg µl^−1^ in PBS) in the heart of 72 hpf larvae using a micromanipulator. Tracer leakage assays were performed by injecting 1 nl of 150,000 Da FITC-labelled dextran (FD150S, 25 μg µl^−1^ in PBS) intracardially and imaging 1 h after injection. Three-dimensional reconstructions were performed using the Imaris Filament Tracer software (Bitplane) before manual false-colouring to highlight extra- and intracerebral vessels exhibiting or not BBB properties.

### Transmission electron microscopy

WT zebrafish embryos (32 hpf) were fixed overnight in 2.5% glutaraldehyde (Electron Microscopy Sciences), 4% PFA at 4 °C and post-fixed with 1% osmium tetroxide (Electron Microscopy Sciences) and 1.5% ferrocyanide (Electron Microscopy Sciences) in 0.15 M cacodylate buffer. The embryos were further stained with 1% uranyl acetate (Electron Microscopy Sciences), serially dehydrated and embedded in epoxy resin (Agar 100 resin; Agar Scientific). Resin blocks containing the processed embryos were trimmed to reach the ROI, which was evaluated by toluidine staining of thin sections (15 μm). Ultrathin 70 nm sections were then produced with a Leica EM UC6 ultramicrotome and mounted onto copper-Formvar-carbon grids (Electron Microscopy Sciences). Observations were made using the Tecnai 10 transmission electron microscope (FEI), and images were captured with a Veleta camera and processed using SIS iTEM v.5.1 software (Olympus).

### Western blot analysis

Samples were denatured in Bolt LDS sample buffer and reducing agent (Thermo Fischer Scientific, B0007 and B0009) at 70 °C for 10 min. Gel electrophoresis was performed using 4–15% Mini-PROTEAN TGX Precast Protein Gels (Bio-Rad, 4561085). Proteins were transferred to nitrocellulose membranes. After blocking in 5% milk in Tris-buffered saline (TBS), the membranes were incubated with primary antibodies (1% milk in 0.05% Tween-20 TBS, TBST) overnight at 4 °C. After washing in TBST, membranes were incubated with secondary antibodies in 1% BSA in TBST, for 1 h at room temperature. Blots were revealed using Western Lightning Plus ECL (PerkinElmer, NEL103001EA).

The following primary antibodies were used: rabbit anti-HA (Merck, H6908, 1:1,000), chicken anti-GFP (Aves Biolabs, GFP-1020, 1:10,000), rat anti-laminin-111 (R&D systems, MAB4656, 1:250, monoclonal reactivity towards LAMA1/B1). The following secondary antibodies were used: goat anti-rabbit IgG HRP conjugate (Promega, W401B, 1:5,000), goat anti-chicken IgY HRP conjugate (Thermo Fischer Scientific, A16054, 1:40,000) and rabbit anti-rat IgG HRP conjugate (Merck, A9542, 1:5,000). Uncropped blots are provided in Supplementary Fig. 1.

### Recombinant protein expression and purification

The human MMP25 and MMP2 catalytic domains were amplified from HUVEC cDNA and the zebrafish Mmp25b catalytic domain was synthesized after codon optimization. The fragments were cloned into the NcoI and XhoI restriction sites of pET21d. The catalytic domains span residues Tyr113 to Gly284 of zebrafish Mmp25b (UniProtKB: E7F1N5), Tyr108 to Gly280 of human MMP25 (UniProtKB: Q9NPA2) and Tyr110 to Asp452 of human MMP2 (UniProtKB: P08253). BL21 (DE3) *E. coli* cells were transformed with pET21d-zMmp25b-6xhis, pET21d-hMMP25-6xhis or pET21d-hMMP2-6xhis and grown in 100–300 ml LB medium (supplemented with 100 µg ml^−1^ ampicillin). Protein expression was induced with 1 mM isopropyl β-d-1-thiogalactopyranoside (IPTG) when the culture reached an optical density at 600 nm (OD_600_) of 0.9. After overnight incubation at 37 °C under agitation, cells were collected by centrifugation (5,000*g*, 20 min, 4 °C) and frozen at −80 °C. After resuspension in 50 mM Tris (pH 8), cells were mechanically lysed on ice (Microfluidics, 110SCE, 3 cycles). Inclusion bodies were recovered from the lysate by centrifugation (16,000*g*, 20 min, 4 °C) and solubilized in 8 M urea, 50 mM Tris (pH 7.6), 150 mM NaCl, 5 mM CaCl_2_ and 50 µm ZnCl_2_. The insoluble fraction was removed by centrifugation (16,000*g*, 20 min, 4 °C) and the supernatant was incubated overnight with 100 µl of Ni^+^/nitrilotriacetic acid agarose beads (Qiagen) at 4 °C. The beads were washed with 20 mM imidazole in TBS 8 M urea and elution was performed with 500 mM imidazole in TBS 8 M urea. Recombinant protein purity was assessed by SDS–PAGE and Coomassie blue staining, and protein concentrations were measured by the BCA protein assay (Thermo Fischer Scientific, 23223). Catalytic domains were refolded by dilution (1/20, v/v) in 50 mM Tris, 150 mM NaCl, 5 mM CaCl_2_, 50 µM ZnCl_2_, 0.005% Brij-35 (Thermo Fischer Scientific, 20150) for 1 h at 12 °C. The insoluble fraction was removed by centrifugation (21,400*g*, 10 min, 4 °C). Uncropped gels are available in Supplementary Fig. 1.

### Mmp25 cleavage assays

For α-1 antitrypsin, 2 µM of α-1 antitrypsin (Athens Research and Technology, 16-16-0011609) was incubated with 2 µM rzMmp25b or 75 nM rhMMP25 overnight at 28 °C and 37 °C, respectively, in 50 µl Mmp25 cleavage buffer (50 mM Tris (pH 7.6), 150 mM NaCl, 5 mM CaCl_2_, 0.005% Brij-35 (Thermo Fischer Scientific, 20150)).

For laminin-111, 15 µg of Matrigel (Corning, 354230) was incubated overnight at 37 °C with 1 µM of rhMMP25 in 50 µl Mmp25 cleavage buffer. The samples were concentrated by acetone precipitation before SDS–PAGE and western blot analysis for LAMA/B1 (R&D systems, MAB4656).

For collagen IV, 20 µg of collagen IV purified from human placenta (Merck, C7521) was incubated with 1 µM rhMMP25 overnight at 37 °C in 50 µl Mmp25 cleavage buffer. The samples were concentrated by acetone precipitation before SDS–PAGE and Coomassie blue staining.

For recombinant HA-tagged Col4a5 expressed in HEK293T cells, Zebrafish *col4a5* was amplified from 48 hpf zebrafish cDNA, cloned in fusion to a C-terminal HA tag into pCS2^+^ (digested with BamHI and XhoI) and transiently expressed using PEI (polyethylenimine) in HEK293T cells (ATCC CRL-3216, authenticated by ATCC STR profiling, tested negative for mycoplasma contamination). The empty pCS2^+^ was used as negative control. Then, 48 h after transduction, the cells were washed twice in PBS, before collection and cell disruption using a disposable grinding pestle in Mmp25 cleavage buffer. After centrifugation (21,400*g*, 10 min, 4 °C), 4 µg of the supernatant was incubated overnight with 2 µM of rzMmp25b at 28 °C or 75 nM of rhMMP25 at 37 °C in 50 µl Mmp25 cleavage buffer.

For human COL4A1–6 putative cleavage sites expressed as GST–GFP linkers in *E.coli*, DNA sequences encoding a N-terminal fusion between a 12 amino acid fragment centred on the putative cleavage site of MMP25 in COL4A1–6 and GFP were cloned into pGEX-6P-1 downstream of the GST and the recognition sequence for site-specific cleavage by the PreScission Protease-encoding sequences. BL21 (DE3) *E. coli* were transformed with these constructs and protein expression was induced with 1 mM IPTG when OD_600_ reached 0.7. After overnight incubation at 30 °C under agitation, cells were collected by centrifugation (5,000*g*, 20 min at 4 °C) and lysed in 50 mM Tris (pH 8) using the FastPrep-24 cell disrupter and Lysing Matrix B Bulk (M.P. Biomedicals). After three cell disruption cycles of 20 s, the cell lysates were clarified by centrifugation (21,400*g*, 10 min, 4 °C). Protein concentration of the supernatant was determined using BCA (Thermo Fischer Scientific, 23223). A total of 500 ng of the soluble fraction was incubated overnight in Mmp25-cleavage buffer with 75 nM of rhMMP25 or rhMMP2 at 37 °C, or with 1 × 10^−3^ IU of the control PreScission Protease (GenScript, N02799-100) at 25 °C in 50 µl Mmp25 cleavage buffer. Uncropped gels and blots are available in Supplementary Fig. 1.

### MS analysis

For protein digestion, bands of interest were excised from SDS–PAGE gels, washed twice with distilled water and shrunk in 100% acetonitrile. In-gel proteolytic digestion was performed by the addition of 4 µl of trypsin (Promega; in 50 mM NH_4_HCO_3_) and overnight incubation at 37 °C.

For MS, protein digests (supernatants) were analysed using nano-liquid chromatography–electrospray ionization–MS/MS on the timsTOF Pro (Bruker v.5.3) system. Peptides were separated by nanoUHPLC (nanoElute, Bruker) on a 75 μm inner diameter, 25 cm C18 column with integrated CaptiveSpray insert (Aurora, IonOpticks) at a flow rate of 200 nl min^−1^, at 50 °C. LC mobile phase A was 0.1% formic acid (v/v) in H_2_O, and mobile phase B was 0.1% formic acid (v/v) in acetonitrile. Digests (1 µl) were loaded at a constant pressure of 600 bar, directly on the column. After injection of the digest (1 µl), the mobile phases were linearly increased from 2% B to 13% over 18 min, from 13% B to 19% over 7 min, from 19% B to 22% over 4 min, and from 22% B to 85% in 3 min.

Data acquisition on the timsTOF Pro was performed using Hystar v.5.1 and timsControl v.2.0. The TIMS accumulation time was 100 ms and mobility (1/*K*_0_) ranged from 0.6 to 1.6 V s cm^−2^. Analyses were performed using parallel accumulation serial fragmentation (PASEF) acquisition method^75^. Per total cycle of 1.1 s, one MS spectrum was followed by ten PASEF MS/MS spectra.

For data processing, tandem mass spectra were extracted, charge-state deconvoluted and deisotoped by Data analysis (Bruker) v.5.3. All MS/MS samples were analysed using Mascot (Matrix Science; v.2.8.1), searching the Human Proteome database (https://www.uniprot.org/uniprotkb?query=(proteome:UP000005640), 101,673 entries) assuming semi-specific trypsin digestion. Three missed cleavages were tolerated. Mascot was searched with a fragment ion mass tolerance of 0.050 Da and a parent ion tolerance of 15 ppm. Carbamidomethyl of cysteine was specified as a fixed modification in Mascot. Oxidation of methionine, hydroxylation of lysine and proline, deamination of asparagine and glutamine, and acetylation of the N-terminus were specified in Mascot as variable modifications.

Peptide and protein identifications were performed using Scaffold (v.Scaffold_5.10.0, Proteome Software). Peptide identifications were accepted by the Scaffold Local FDR algorithm if establishing a probability higher than 96.0% to achieve an FDR lower than 1.0%. Protein identifications were accepted if the probability was higher than 5.0% to achieve an FDR lower than 1.0% and containing at least two identified peptides. Protein probabilities were assigned by the Protein Prophet algorithm^76^. Proteins that contained similar peptides and could not be differentiated based on MS/MS analysis alone were grouped to satisfy the principles of parsimony. Proteins sharing significant peptide evidence were grouped into clusters.

### Statistics and reproducibility

Seurat v.4 was used to analyse the scRNA-seq datasets. Bulk RNA-seq data were analysed using DESeq2 v.1.12. Statistical analyses were performed using RStudio v.1.1.463 and GraphPad Prism v.9. Pearson correlation analyses and visualizations were performed using ggcorrplot v.0.1.3. Normally distributed data are represented as mean ± s.d. and were analysed using one-tailed one-way ANOVA (with post hoc Dunnett’s test) and two-tailed Student’s *t*-tests for multiple and single comparisons, respectively. Non-normally distributed data are represented as median ± interquartile range and were analysed using one-tailed Kruskal–Wallis tests (with post hoc Dunn’s test) for multiple comparisons and two-tailed Mann–Whitney *U*-tests for single comparisons. No statistical methods were used to determine the sample size. The sample size was determined by the technical constraints of the experiments, as well as our and other’s previous work on zebrafish neurovascular development^11–14,17,71^. One-cell stage embryos are undistinguishable irrespective of their genotype, and were therefore randomized during injections. The allocation of organisms into experimental groups was randomized. Experimental groups of an experiment were always raised in parallel, under identical conditions. For zebrafish and mouse Mendelian genetics experiments, genotyping was always performed after phenotypic assessment. The researcher is therefore inherently blinded to the experimental conditions. In MO and somatic gene disruption experiments, investigators were not blinded. The sex of animals was not determined (embryonic or larval zebrafish) or was not analysed (embryonic mice) at the developmental stage of interest. The number and nature of observations (*n*), mean or median, type of error bar and statistical tests used for analysis are indicated in the figure legends. Images of immunofluorescence, in situ hybridization, transmission electron microscopy, and protein gels or blots are representative of experiments that were repeated independently at least three times. All attempts at replication were successful.

### Reporting summary

Further information on research design is available in the Nature Portfolio Reporting Summary linked to this article.

## Online content

Any methods, additional references, Nature Portfolio reporting summaries, source data, extended data, supplementary information, acknowledgements, peer review information; details of author contributions and competing interests; and statements of data and code availability are available at 10.1038/s41586-024-07283-6.

### Supplementary information

Supplementary Fig. 1 Raw, unprocessed images of Coomassie blue gels and western blots relating to Fig. 3j,k,m and Extended Data Figs. 8c–e and 10f.

Reporting Summary

Supplementary Video 1 Brain vascular invasion is initiated by Wnt–β-catenin signalling-competent TCs in genetically mosaic zebrafish.

Supplementary Video 2 Intraneural vessels can be guided by Wnt–β-catenin signalling-incompetent TCs in genetically mosaic zebrafish.

Supplementary Video 3 PHBCs contain Wnt–β-catenin signalling-positive and -negative ECs, while CNS-invading TCs are always positive.

Supplementary Video 4 Wnt–β-catenin signalling is dispensable for VEGF-dependent endothelial Ca^2+^ oscillations in the perineural endothelium.

Supplementary Video 5 Intravital imaging of hindbrain angiogenesis in WT and *mmp25a/b*^*−/−*^ zebrafish embryos.

Supplementary Video 6 Intravital imaging of intersegmental vessel formation formation in WT and *mmp25a/b*^*−/−*^ zebrafish embryos.

Supplementary Video 7 Mmp25 is dispensable for VEGF-dependent endothelial Ca^2+^ oscillations in the perineural endothelium.

Supplementary Video 8 *mmp25a/b*^*−/−*^ ECs are outcompeted by WT cells during brain angiogenesis.

### Source data

Source Data Fig. 1

Source Data Fig. 2

Source Data Fig. 3

Source Data Fig. 4

Source Data Extended Data Fig. 1

Source Data Extended Data Fig. 2

Source Data Extended Data Fig. 3

Source Data Extended Data Fig. 4

Source Data Extended Data Fig. 5

Source Data Extended Data Fig. 6

Source Data Extended Data Fig. 7

Source Data Extended Data Fig. 8

Source Data Extended Data Fig. 10

## Data Availability

The RNA-seq and MS data were deposited at the NCBI Gene Expression Omnibus through GEO Series accession numbers GSE121041, GSE233488 and GSE233662 and in PRIDE with the dataset identifier PXD042613 (Proteomics Identification Database), respectively. Databases used in this study include UniProt (E7F1N5, Q9NPA2 and P08253). The Human Proteome database (https://www.uniprot.org/uniprotkb?query=(proteome:UP000005640)) was used for MS/MS analysis. Publicly accessible datasets used in this study are: ref. ^40^, (Sequence Read Archive: PRJNA637987 and http://mousebrain.org/); ref. ^42^ (GEO: GSE150219); ref. ^44^ (GEO: GSE122564); ref. ^77^ (GEO: GSE79306); ref. ^78^ (GEO: GSE111839); ref. ^79^ (GEO: GSE95401 and GSE95201); ref. ^80^ (GEO: GSE52564); ref. ^81^ (GEO: GSE66848); ref. ^82^ (GEO: GSE74052). All other data are available in the Article and its Supplementary Information.  Source data are provided with this paper.
