# Supplementary material for: A brain-specific angiogenic mechanism enabled by tip cell specialization
Source: Nature. 2024 Apr 3;628(8009):863–71. doi: 10.1038/s41586-024-07283-6 (PMC11041701; doi:10.1038/s41586-024-07283-6)

nature portfolio

https://doi.org/10.1038/s41586-024-07283-6

Supplementary information

A brain-specific angiogenic mechanism enabled by tip cell specialization

In the format provided by the authors and unedited

Related to Figure 3j

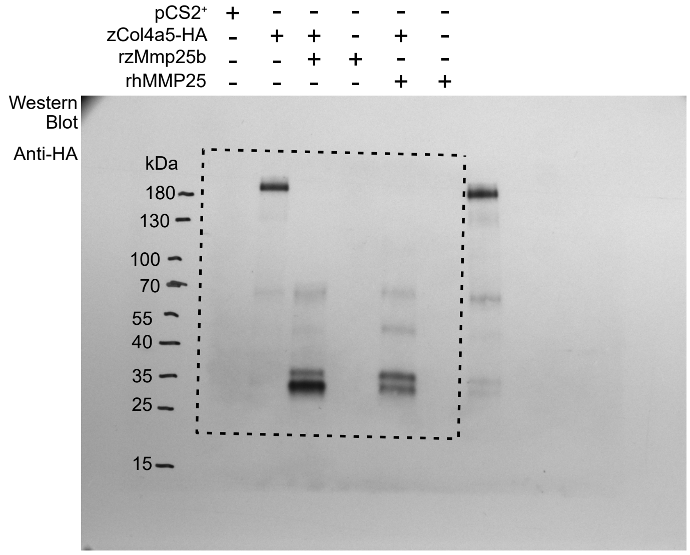

Related to Figure 3k

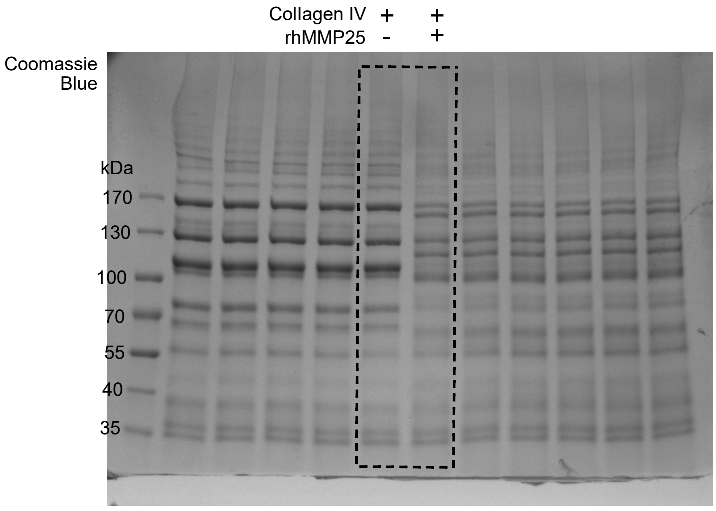

Related to Figure 3m

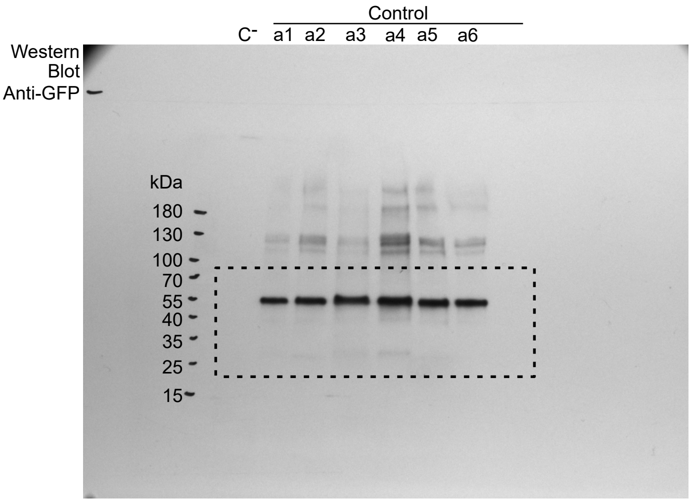

Related to Figure 3m

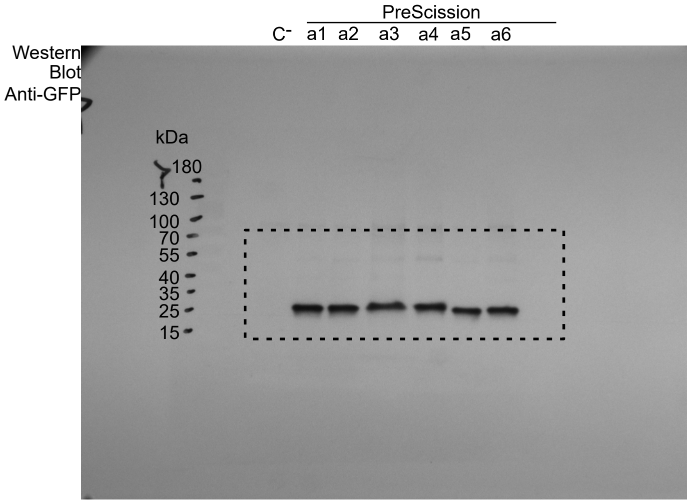

Related to Figure 3m

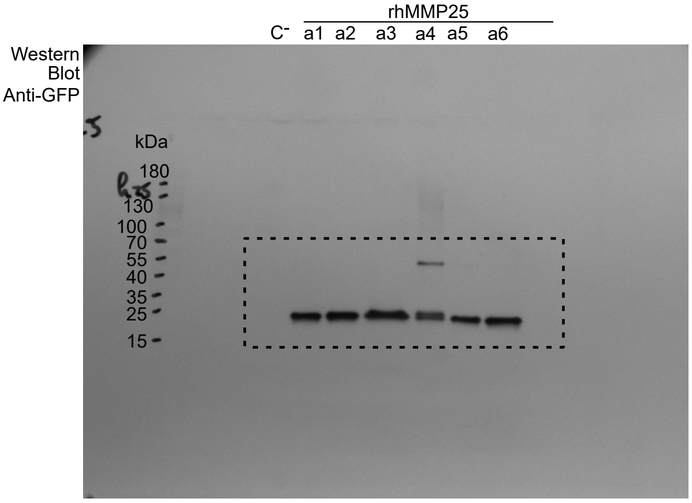

Related to Figure 3m

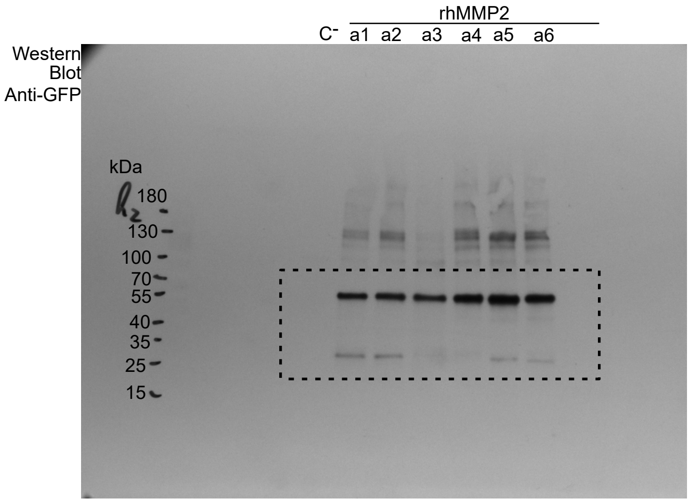

Related to Extended data Figure 8c

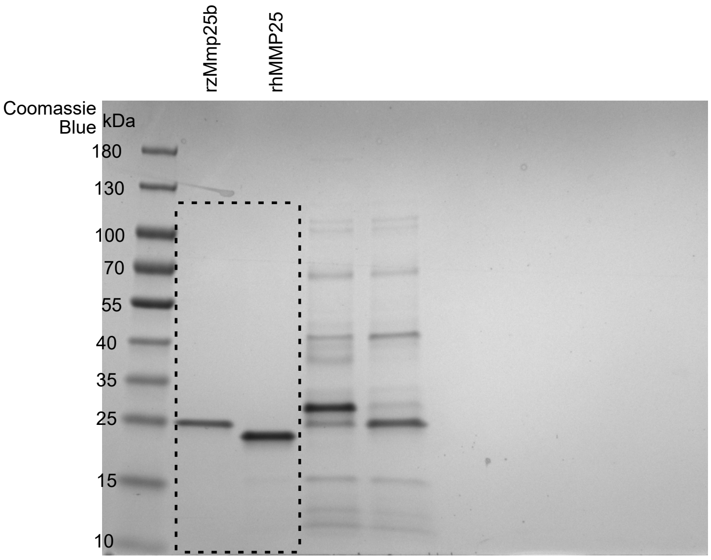

Related to Extended data Figure 8d

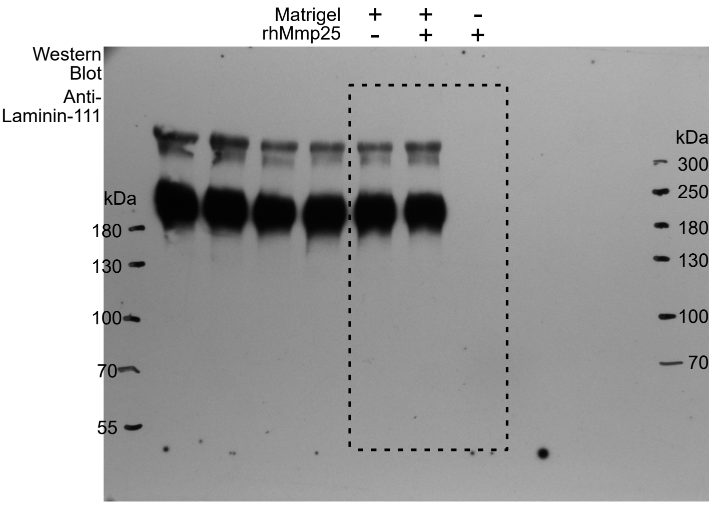

Related to Extended data Figure 8e

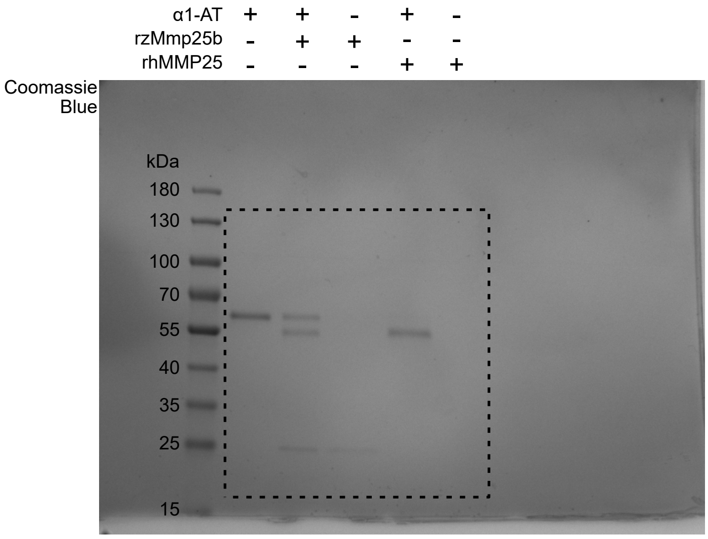

Related to Extended data Figure 10f

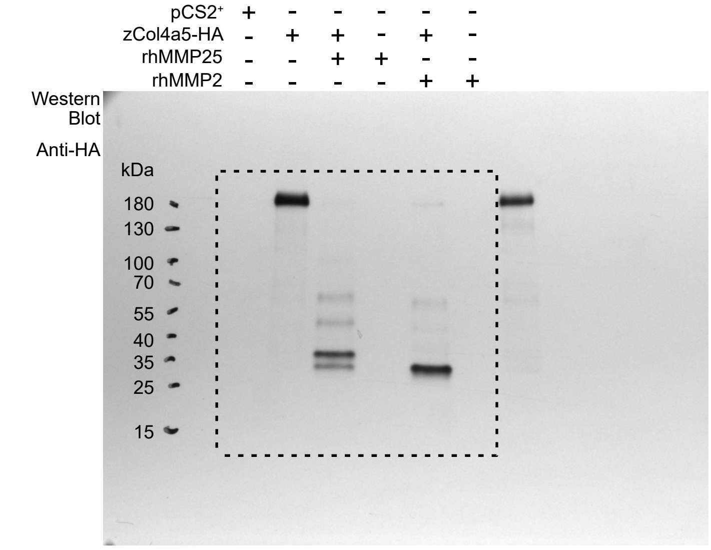

Supplement: Supplementary file 1 — Raw, unprocessed images of Coomassie blue gels and western blots relating to Fig. 3j,k,m and Extended Data Figs. 8c–e and 10f. [file 41586_2024_7283_MOESM1_ESM.pdf]
