# Supplementary material for: A brain-specific angiogenic mechanism enabled by tip cell specialization
Source: Nature. 2024 Apr 3;628(8009):863–71. doi: 10.1038/s41586-024-07283-6 (PMC11041701; doi:10.1038/s41586-024-07283-6)
Supplement: Supplementary file 2 — Reporting Summary [file 41586_2024_7283_MOESM2_ESM.pdf]

nature portfolio

Corresponding author(s): Benoit Vanhollebeke

Last updated by author(s): Feb 11, 2024

Reporting Summary

Nature Portfolio wishes to improve the reproducibility of the work that we publish. This form provides structure for consistency and transparency in reporting. For further information on Nature Portfolio policies, see our Editorial Policies and the Editorial Policy Checklist.

Statistics

For all statistical analyses, confirm that the following items are present in the figure legend, table legend, main text, or Methods section.

n/a	Confirmed	
☐	☒	The exact sample size (*n*) for each experimental group/condition, given as a discrete number and unit of measurement
☐	☒	A statement on whether measurements were taken from distinct samples or whether the same sample was measured repeatedly
☐	☒	The statistical test(s) used AND whether they are one- or two-sided<br>*Only common tests should be described solely by name; describe more complex techniques in the Methods section.*
☒	☐	A description of all covariates tested
☒	☐	A description of any assumptions or corrections, such as tests of normality and adjustment for multiple comparisons
☐	☒	A full description of the statistical parameters including central tendency (e.g. means) or other basic estimates (e.g. regression coefficient) AND variation (e.g. standard deviation) or associated estimates of uncertainty (e.g. confidence intervals)
☐	☒	For null hypothesis testing, the test statistic (e.g. *F*, *t*, *r*) with confidence intervals, effect sizes, degrees of freedom and *P* value noted<br>*Give P values as exact values whenever suitable.*
☒	☐	For Bayesian analysis, information on the choice of priors and Markov chain Monte Carlo settings
☒	☐	For hierarchical and complex designs, identification of the appropriate level for tests and full reporting of outcomes
☐	☒	Estimates of effect sizes (e.g. Cohen's *d*, Pearson's *r*), indicating how they were calculated

Our web collection on statistics for biologists contains articles on many of the points above.

Software and code

Policy information about availability of computer code

Data collection	ImageJ (Fiji) 1.53c, ZEN Blue 3.1, ZEN Black 2.1 SP3, Leica Application Suite (LAS) v4.2, Hystar v5.1 and timsControl v2.0.
Data analysis	Data analysis software used in this study includes Graphpad prism v9, Jalview v2.11.3.2, Rstudio v1.1.463, Bruker v5.3, Mascot v2.8.1, Scaffold_5.10.0 and SIS iTEM v5.1 sofware. The Protein Prophet algorithm was used for mass spectrometry analysis. R packages used in this study include Seurat v4, DESeq2 v1.12, ggcorrplot v0.1.3. The CRISPOR tool (v5.01) was used to determine sgRNA target sites. Morpholino target sequences were determined by Gene Tools (Eugene, OR). The Illumina pipeline (bcl2fastq v2.19.0.316), Nextera XT index kit v2 adapters, the TopHat v2.1.1 tool (Bowtie1 or Bowtie2 option), Trim Galore v0.4.4, Samtools v1.16.1 and the Subread package v1.4.6-p5 were used for RNA-seq data processing. BioRender and Imaris Filament Tracer software (Bitplane) were used for data visualisation.

For manuscripts utilizing custom algorithms or software that are central to the research but not yet described in published literature, software must be made available to editors and reviewers. We strongly encourage code deposition in a community repository (e.g. GitHub). See the Nature Portfolio guidelines for submitting code & software for further information.

Data

Policy information about availability of data

All manuscripts must include a data availability statement. This statement should provide the following information, where applicable:

- Accession codes, unique identifiers, or web links for publicly available datasets
- A description of any restrictions on data availability
- For clinical datasets or third party data, please ensure that the statement adheres to our policy

The RNA-Seq and mass spectrometry data were deposited in NCBI's Gene Expression Omnibus through GEO Series accession numbers GSE121041, GSE233488, and GSE233662 and in PRIDE with the dataset identifier PXD042613 and 10.6019/PXD042613 (Proteomics Identification Database), respectively.

Databases used in this study include Uniprot (https://www.uniprot.org/), accession numbers UniProtKB:E7F1N5, UniProtKB:Q9NPA2, and UniProtKB:P08253. The Human Proteome database https://www.uniprot.org/uniprotkb?query=(proteome:UP000005640) was used for MS/MS analysis.

Publicly accessible datasets used in this study are:
La Manno et al., 2021, ref. 40, (Sequence Read Archive: accession PRJNA637987 and http://mousebrain.org/)
DeSisto et al., 2020, ref. 42, (NCBI Gene Expression Omnibus: accession GSE150219)
Corada et al., 2019, ref. 44, (NCBI Gene Expression Omnibus: accession GSE122564)
Hupe et al., 2017, ref. 77, (NCBI Gene Expression Omnibus: accession GSE79306)
Sabbagh et al., 2018, ref. 78, (NCBI Gene Expression Omnibus: accession GSE111839)
Munji et al., 2019, ref. 79, (NCBI Gene Expression Omnibus: accessions GSE95401 and GSE95201)
Zhang et al., 2014, ref. 80, (NCBI Gene Expression Omnibus: accession GSE52564)
Jensen et al., 2019, ref. 81, (NCBI Gene Expression Omnibus: accession GSE66848)
Chang et al., 2017, ref. 82, (NCBI Gene Expression Omnibus: accession GSE74052)

All other data are available in the main text, Extended data, Supplementary information, or Source data. Correspondence and requests for materials should be addressed to B.V.

Research involving human participants, their data, or biological material

Policy information about studies with human participants or human data. See also policy information about sex, gender (identity/presentation), and sexual orientation and race, ethnicity and racism.

Reporting on sex and gender	N/A
Reporting on race, ethnicity, or other socially relevant groupings	N/A
Population characteristics	N/A
Recruitment	N/A
Ethics oversight	N/A

Note that full information on the approval of the study protocol must also be provided in the manuscript.

Field-specific reporting

Please select the one below that is the best fit for your research. If you are not sure, read the appropriate sections before making your selection.

☒ Life sciences ☐ Behavioural & social sciences ☐ Ecological, evolutionary & environmental sciences

For a reference copy of the document with all sections, see nature.com/documents/nr-reporting-summary-flat.pdf

Life sciences study design

All studies must disclose on these points even when the disclosure is negative.

Sample size	No statistical methods were used to determine sample size. Sample size was determined by the technical constraints of the experiments, as well as our and other's previous work on zebrafish neurovascular development.
Data exclusions	In single-cell RNA sequencing experiments, to ascertain the inclusion of living and deeply sequenced cells, we used the following pre-established criteria: Cells were used only when in accordance with the following criteria: The number of different transcripts detected was higher than 4,000, the total number of counts was higher than 190,000 and the contribution of mitochondrial DNA was lower than 1.8% (indicating cell death). Transcripts were included when expressed in at least three cells and detected at least 200 times over all samples. In mass spectrometry analysis, to ensure proper peptide and protein identifications, the following pre-established criteria were used: peptide identifications were accepted by the Scaffold Local FDR algorithm if establishing a probability higher than 96.0% to achieve an FDR lower than

	1.0%. Protein identifications were accepted if the probability was higher than 5.0% to achieve an FDR lower than 1.0% and containing at least two identified peptides. No other data points or samples were excluded from the analysis in this study.
Replication	The number and nature of observations (n), mean or median, type of error bar, and statistical tests used for analysis are indicated in the figure legends. Images of immunofluorescence, in situ hybridization, transmission electron microscopy, and protein gels or blots are representative of experiments that were repeated independently, at least 3 times. All attempts at replication were successful.
Randomization	One-cell stage embryos are undistinguishable irrespective of their genotype, and were thus randomized during injections. The allocation of organisms into experimental groups was randomized. Experimental groups of an experiment were always raised in parallel, under identical conditions.
Blinding	For zebrafish and mouse Mendelian genetics experiments, genotyping was always performed after phenotypic assessment. The researcher is thus inherently blinded to the experimental conditions. In morpholino and somatic gene disruption experiments, investigators were not blinded.

Reporting for specific materials, systems and methods

We require information from authors about some types of materials, experimental systems and methods used in many studies. Here, indicate whether each material, system or method listed is relevant to your study. If you are not sure if a list item applies to your research, read the appropriate section before selecting a response.

Materials & experimental systems

n/a	Involved in the study
☐	☒ Antibodies
☐	☒ Eukaryotic cell lines
☒	☐ Palaeontology and archaeology
☐	☒ Animals and other organisms
☒	☐ Clinical data
☒	☐ Dual use research of concern
☒	☐ Plants

Methods

n/a	Involved in the study
☒	☐ ChIP-seq
☒	☐ Flow cytometry
☒	☐ MRI-based neuroimaging

Antibodies

Antibodies used

Primary antibodies: Rabbit anti-HA (Merck, H6908, polyclonal, lot: 0000126958), chicken anti-GFP (Aves Biolabs, GFP-1020, polyclonal, lot: GFP3717982), rat anti-Laminin-111 (R&D systems, MAB4656, monoclonal clone AL-4, Lot: ZZG0219091), rabbit anti-Laminin-111 (Merck, L9393, polyclonal, lot: 0000082508), rabbit anti-Collagen type IV (Sigma-Aldrich, AB756P, polyclonal, lot: 3607063), chicken Anti-β-Galactosidase (Abcam, ab9361, polyclonal, lot: GR257143-2), rabbit anti-Erg-Alexa Fluor (AF) 647 conjugate (Abcam, ab196149, monoclonal clone EPR3864, lot: GR3398432-4), sheep anti-DIG-alkaline phosphatase conjugate (Merck, 11093274910, polyclonal, lot: 11266026), sheep anti-DIG horseradish peroxidase conjugate (Merck, 11207733910, polyclonal, lot: 64012000).

Secondary antibodies: Goat anti-rabbit IgG HRP conjugate (Promega, W401B, polyclonal, lot: 0000536252), goat anti-chicken IgY HRP conjugate (Thermo Fischer Scientific, A16054, polyclonal, lot: 80-173-021622), rabbit anti-rat IgG HRP conjugate (Merck, A9542, polyclonal, lot: 045M4819V), goat anti-chicken AF488 (Thermo Fischer Scientific, polyclonal, A11039, lot: 1458638), goat anti-rabbit AF594 (Thermo Fischer Scientific, A11012, polyclonal, lot: 2165334), and donkey anti-rat AF647 (Thermo Fischer Scientific, A48272, polyclonal, lot: XF348188).

Validation

Rabbit anti-HA (Merck, H6908): Polyclonal. Anti-HA antibody is specific for N- or C-terminal HA-tagged fusion proteins. Immunogen: Synthetic peptide corresponding to amino acid residues of human Influenza virus hemagglutinin (HA) known as HA-tag, conjugated to KLH. The antibody is affinity-purified on the immobilized immunizing peptide. The immunizing HA peptide (Product No.I2149) specifically inhibits the staining of the HA-tagged protein band. Fig.4j shows immunoreactivity to HEK293T protein extracts from cells overexpressing HA-tagged zCol4a5 fusion proteins and not in negative controls (transfected with pCS2+).

Chicken anti-GFP (Aves Biolabs, GFP-1020): Polyclonal. Immunogen: Recombinant GFP expressed in Escherichia coli. Affinity-purified antibodies were prepared using GFP conjugated to an agarose matrix. Fig. 4m shows immunoreactivity to BL21 (DE3) E. coli protein extracts, in which GST-GFP fusion proteins were overexpressed and not in negative controls (transformed with GST).

Rat anti-Laminin-111 (R&D systems, MAB4656, Mouse Laminin alpha 1/beta 1 Antibody): Monoclonal Clone AL-4. Immunogen: Purified fragment of chymotrypsin-digested mouse Englebreth Holm-Swarm (EHS) tumor-derived Laminin-1. This antibody has been used to detect Lama1 in mice in previous studies PMID: 32322056, PMID: 28325301.

Rabbit anti-Laminin-111 (Merck, L9393): Polyclonal. Immunogen: Laminin isolated from the basement membrane of Englebreth Holm-Swarm (EHS) mouse sarcoma. This antibody has been used to detect Laminin-111 in zebrafish embryos in previous studies: PMID: 16973147, PMID: 27634568, PMID: 12070089.

Rabbit anti-Collagen type IV (Sigma-Aldrich, AB756P): polyclonal. Immunogen: Collagen Type IV extracted and purified from mouse tumor tissues. Specificity: Antibody shows less than 0.1% reactivity with human Collagen types IV, and V, mouse Collagen Types I, II, and III, mouse fibronectin, and mouse laminin. Species Reactivity Note: Mouse Collagen, Type IV: 100 % (at a 1:5000 RIA dilution) Mouse Collagen, Types I, II, III: <0.1 Human Collagen, Types IV, V: <0.1 Mouse Fibronectin: <0.1 Mouse Laminin: <0.1 Reactivity with

other species has not been determined. This antibody has been used to detect Collagen type IV in mice in previous studies: PMID: 19085956, PMID: 26494538.

Chicken anti-β-Galactosidase (Abcam, ab9361): Polyclonal. Immunogen: The immunogen was purified beta-galactosidase from Escherichia coli. Antibodies were solid phase absorbed then immunoaffinity purified using purified beta-galactosidase immobilized on a solid phase. This antibody has been used to detect β-Galactosidase in mice in previous studies: PMID: 35524138.

Rabbit anti-Erg-Alexa Fluor (AF) 647 conjugate (Abcam, ab196149): Monoclonal EPR3864. This antibody has been used to detect Erg in mice in previous studies, and co-stained cells binding Isolectin B4: PMID: 31043605, PMID: 35571675.

Sheep anti-DIG-alkaline phosphatase conjugate (Merck, 11093274910): Polyclonal. The antibody is specific to digoxigenin and digoxin. Cross reactivity to digitoxin and digitoxigenin: <1 %. No cross reactivity with other human estrogen or androgen steroids, e.g. estradiol or testosterone. After immunization with digoxigenin, sheep IgG was purified by ion-exchange chromatography, and the specific IgG was isolated by immunosorption.

Sheep anti-DIG horseradish peroxidase conjugate (Merck, 11207733910). Polyclonal. The antibody is specific to digoxigenin and digoxin and shows no cross-reactivity with other steroids, such as human estrogens and androgens. After immunization with digoxigenin, sheep IgG was purified by ion-exchange chromatography, and the specific IgG was isolated by immunosorption.

Eukaryotic cell lines

Policy information about cell lines and Sex and Gender in Research

Cell line source(s)	ATCC CRL-3216 (HEK293T).
Authentication	ATCC STR profiling.
Mycoplasma contamination	HEK293T cells tested negative for mycoplasma contamination.
Commonly misidentified lines (See ICLAC register)	No commonly misidentified line was used in the study.

Animals and other research organisms

Policy information about studies involving animals; ARRIVE guidelines recommended for reporting animal research, and Sex and Gender in Research

Laboratory animals	Zebrafish (Danio rerio), in both the AB or TL background were used during, embryonic (0 to 48 hpf) and larval stages (3 to 5 dpf). The exact developmental stage is detailed for every experiment. Transgenic and mutant zebrafish lines used in this study are: Tg(kdrl:EGFP)s843, Tg(kdrl:ras-mCherry)s896, Tg(7xTCF-Xla.Siam:GFP)ia4, Tg(fli1a:Gal4FF)ubs3, Tg(UAS:Kaede)rk8, Tg(UAS:GCaMP7a)zf415, Tg(gata1:DsRed)sd2, gpr124s984 , wnt7aaulb2 , reckulb3 , kdrlhu5088 , and col4a5s510, mmp25aulb26 and mmp25bulb27.<br><br>Mice (Mus musculus), in the C57BL/6J background were used during embryonic stages (E10.5 and E12.5). The mouse lines used in this study are the BAT-GAL reporter (B6.Cg-Tg(BAT-LacZ)3Picc/J) mice and Mmp25 knock-out mice.
Wild animals	The study did not involve wild animals.
Reporting on sex	Sex of animals is not determined (embryonic or larval zebrafish) or was not analyzed (embryonic mice) at the developmental stage of interest.
Field-collected samples	The study did not involve samples collected from the field.
Ethics oversight	Zebrafish and mice were housed in a certified animal facility (LA1500474) in accordance with European and national ethical and animal welfare guidelines. All animal procedures were approved by the corresponding ethical committee (Commission d'Ethique et du Bien Être Animal (CEBEA), Université libre de Bruxelles. Protocol approval numbers are CEBEA-IBMM-2016:65 and CEBEA-07 GOS IBMM for zebrafish, and CEBEA-08 GOS IBMM for mice.

Note that full information on the approval of the study protocol must also be provided in the manuscript.

Plants

Seed stocks	*Report on the source of all seed stocks or other plant material used. If applicable, state the seed stock centre and catalogue number. If plant specimens were collected from the field, describe the collection location, date and sampling procedures.*
Novel plant genotypes	*Describe the methods by which all novel plant genotypes were produced. This includes those generated by transgenic approaches, gene editing, chemical/radiation-based mutagenesis and hybridization. For transgenic lines, describe the transformation method, the number of independent lines analyzed and the generation upon which experiments were performed. For gene-edited lines, describe the editor used, the endogenous sequence targeted for editing, the targeting guide RNA sequence (if applicable) and how the editor was applied.*
Authentication	*Describe any authentication procedures for each seed stock used or novel genotype generated. Describe any experiments used to assess the effect of a mutation and, where applicable, how potential secondary effects (e.g. second site T-DNA insertions, mosiacism, off-target gene editing) were examined.*
